# Comparative Study of Secreted Proteins, Enzymatic Activities of Wood Degradation and Stilbene Metabolization in Grapevine Botryosphaeria Dieback Fungi

**DOI:** 10.3390/jof7070568

**Published:** 2021-07-16

**Authors:** Clément Labois, Elodie Stempien, Justine Schneider, Christine Schaeffer-Reiss, Christophe Bertsch, Mary-Lorène Goddard, Julie Chong

**Affiliations:** 1 Laboratoire Vigne, Biotechnologies et Environnement (LVBE, UPR 3991), Université de Haute Alsace, 68000 Colmar, France; clement.labois@uha.fr (C.L.); elodie.stempien@gmail.com (E.S.); christophe.bertsch@uha.fr (C.B.); 2 Laboratoire d’Innovation Moléculaire et Applications, Université de Haute-Alsace, Université de Strasbourg, CNRS, LIMA, UMR 7042, CEDEX, 68093 Mulhouse, France; 3 Laboratoire de Spectrométrie de Masse BioOrganique (LSMBO), IPHC, Université de Strasbourg, CNRS, UMR7178, 25 Rue Becquerel, 67087 Strasbourg, France; j.schneider@inoviem.com (J.S.); christine.schaeffer@unistra.fr (C.S.-R.)

**Keywords:** *Botryosphaeriaceae*, grapevine, stilbene metabolization, secreted proteins

## Abstract

*Botryosphaeriaceae* fungi are plant pathogens associated with Botryosphaeria dieback. To better understand the virulence factors of these fungi, we investigated the diversity of secreted proteins and extracellular enzyme activities involved in wood degradation and stilbene metabolization in *Neofusicoccum* *parvum* and *Diplodia* *seriata*, which are two major fungi associated with grapevine B. dieback. Regarding the analysis of proteins secreted by the two fungi, our study revealed that *N*. *parvum*, known to be more aggressive than *D*. *seriata*, was characterized by a higher quantity and diversity of secreted proteins, especially hydrolases and oxidoreductases that are likely involved in cell wall and lignin degradation. In addition, when fungi were grown with wood powder, the extracellular laccase and Mn peroxidase enzyme activities were significantly higher in *D*. *seriata* compared to *N.* *parvum*. Importantly, our work also showed that secreted *Botryosphaeriaceae* proteins produced after grapevine wood addition are able to rapidly metabolize the grapevine stilbenes. Overall, a higher diversity of resveratrol and piceatannol metabolization products was found with enzymes of *N. parvum* compared to *D. seriata.* This study emphasizes the diversity of secreted virulence factors found in B. dieback fungi and suggests that some resveratrol oligomers produced in grapevine wood after pathogen attack could be formed via pathogenic fungal oxidases.

## 1. Introduction

Grapevine (*Vitis vinifera* L.) is a very important economic crop throughout the world, but it is susceptible to a wide range of pathogens. In the last decades, French and worldwide vineyards have been facing the explosion of grapevine trunk diseases (GTDs), especially Eutypa dieback (E. dieback), esca, and Botryosphaeria dieback (B. dieback) [1]. GTDs are responsible of a dramatic impact on viticulture sustainability and wine production, causing huge losses estimated to exceed over 1 billion dollars per year [2]. Unfortunately, to date, no efficient treatment exists to prevent, protect, or limit the progression of these diseases. One exception, related to esca disease, concerns the application of a foliar fertilizer mixture of calcium, magnesium, and seaweed (Algescar^®^, Natural Development Group, Castelmaggiore, Bologna, Italy), which is able to significantly reduce the foliar symptoms and consequently the loss in grape quantity and quality [3]. GTDs are associated with the growth of xylem-inhabiting fungi complexes, which eventually leads to the sudden death of the plant. They are characterized by a latent state where fungi are present in the plant as endophytes, and a pathogenic state where they become virulent and colonize the vine wood tissues [4,5]. GTDs are likely caused by a combination of biotic and abiotic factors whose implication is not completely known. Many symptoms can be associated to the development of GTDs such as sectorial and/or central necrosis in woody tissues, leaf discoloration, and wilting of berries and inflorescences, leading in the long term to the death of the plant [6,7,8,9,10].

Many fungi have been reported to be involved in GTDs, mainly Eutypa lata (*E. lata*) for E. dieback, and Phaeomoniella chlamydospora (P. chlamydospora), Phaeoacremonium minimum (P. minimum), and Fomitiporia mediterranea (F. mediterranea) for esca [4,11,12,13]. B. dieback is associated with a large number of Botryosphaeriaceae fungal species. Twenty-six different Botryosphaeriaceae species have been found in vineyards of several countries, but Lasiodiplodia theobromae (L. theobromae), Neofusicoccum parvum (N. parvum), and Diplodia seriata (D. seriata) are the most frequently associated with this disease [4].

The main virulence factors of GTD fungi are represented by toxin production and wood decay enzyme synthesis. It has been well documented that fungi involved in GTDs produce a wide variety of toxic secondary metabolites considered as toxins [12,14,15,16]. Since GTDs pathogens are invariably present in the wood but have never been isolated from the leaves of infected plant, it has been suggested that the foliar symptoms could be the consequence of toxin synthesis in the wood. Phytotoxic compounds are supposed to be translocated to the leaves by the transpiration flux [12].

Concerning E. dieback, *E. lata* is able to produce a large number of secondary metabolites, mainly heterocyclic and acetylenic compounds. Among all the toxic compounds synthesized by *E. lata*, only one compound, eutypin, appears to be truly toxic after studies conducted on excised grape leaves and protoplasts [17]. The three main fungi associated with esca produce phytotoxic metabolites including several napthalenone pentaketides. Nine, eight, and three toxic compounds were found in *P. chlamydospora*, *P. minimum,* and *F. mediterranea* culture medium, respectively [14,18]. Moreover, *P. chlamydospora* and *P. minimum* are able to produce pullulan, which is an exopolysaccharide (EPS). When pullulan is directly injected in young shoots, Bruno and Sparapano [19] observed the same foliar symptoms as those observed in the vineyard [20]. Concerning B. dieback, *N. parvum* and *D. seriata* are able to produce numerous compounds belonging to different chemical classes: naphtalenones, naphtoquinones, dihydrotoluquinones, epoxylactones, dihydroisocoumarins, hydroxybenzoic acids, fatty esters, and phenolic compounds (see [16,21] for a review). In addition, a study conducted by Martos et al. showed the production of high molecular weight EPS in five *Botryosphaeriaceae* isolated from grapevine on the decline in Spain. Bénard-Gellon et al. also reported that secreted proteins from *N. parvum* were able to induce necrosis on *Vitis vinifera* cv. Chardonnay cells [22].

In addition to phytotoxin production, another virulence factor of GTD fungi is their ability to degrade grapevine wood. Several authors have reported that pathogens related to GTDs are able to produce wood-degrading enzymes to gain nutrients and expand in the trunk. *E. lata* synthesizes wood-degrading enzymes such as cellulases, chitinases, glucanases, glycosidases, phenol oxidases, polygalacturonases, proteases, xylanases, and starch-degrading enzymes [23,24,25]. It was also discovered that wood degradation by *P*. *chlamydospora* and *P. minimum* is performed thanks to amylases, cellulases, laccases, lipases, pectate lyases, polygalacturonases, proteases, and xylanases [20,26,27]. In 2014, Esteves et al. showed the synthesis of extracellular enzymes in a group of 56 *Botryosphaeriaceae* including *D. seriata* and *N. parvum*. They highlighted the production of amylases, cellulases, laccases, lipases, pectinases, pectin lyases, and proteases [26]. A recent study has also shown an expansion of specific genes involved in cell wall degradation in the genome of fungi involved in GTDs, including *N. parvum* and *D. seriata* [27].

It is known that abiotic and/or biotic stresses will trigger in the plant the activation of defense mechanisms such as phytoalexin biosynthesis [28,29]. In grapevine, phytoalexins belong to the stilbene family and are characterized by a 1,2-diphenylethylene backbone [30]. In vine plants, the stilbene family is very large, and resveratrol is the first stilbene synthesized by the phenylpropanoid pathway in a similar way as flavonoids [31]. Other stilbenoid compounds are obtained after different modifications of resveratrol such as methylation, glycosylation, prenylation, or oligomerization [30]. Piceid (glycosylated resveratrol) is a non-toxic storage form, while stilbene dimers δ-viniferin, ε-viniferin, and methoxylated resveratrol (pterostilbene) are highly toxic. δ-viniferin, obtained by the oxidative dimerization of resveratrol, was described as one of the major stilbenes occurring in UV-stressed grapevine leaves by Pezet et al. [32]. In the case of grapevine trunk diseases, the accumulation of stilbenes (resveratrol and derivatives) was described in several organs of grapevine affected by esca [33,34,35,36,37]. These compounds were particularly higher in symptomatic leaves of esca-infected vines. Given the absence of pathogens in the leaves, it was suggested that foliar symptoms expression could be due to plant response in terms of hypersensitivity reaction, which is triggered by toxic metabolites that reach the canopy from infected wood through the transpiration stream and is usually followed by the subsequent synthesis of phytoalexins [34]. In a previous work, we showed an accumulation of multimeric stilbene forms (dimers, trimers, and tetramers) 7 days after the inoculation of detached grapevines canes by *N. parvum* [38]. In addition, genes encoding PAL (phenylalanine ammonia lyase) and stilbene synthase (STS) were strongly expressed in asymptomatic leaves before the appearance of the esca apoplectic form [39]. Stempien et al. also showed an activation of *VvSTS1* expression as well as an increase in δ-viniferin together with a decrease in resveratrol and piceid contents in grapevine cell cultures treated with extracellular proteins isolated from *N. parvum* and *D. seriata*. A decrease in resveratrol and piceid could be due to stilbene metabolization by fungi or by their use for the synthesis of oligomeric forms [40].

Despite the induction of several defense mechanisms in the wood, especially stilbene synthesis, GTD-associated fungi are able to colonize grapevine trunk. In a previous work, Stempien et al. showed that *N. parvum* and *D. seriata* were able to degrade the carbohydrate components of the cell wall via cellulase and hemicellulase activities. In addition, laccase and Mn peroxidase activities involved in wood decay were measured in the culture medium of both fungi, especially when supplemented with grapevine sawdust. Interestingly, laccase and Mn peroxidase activities were significantly higher for *D. seriata* compared to *N. parvum*. Stempien et al. further evidenced that these fungi are able to efficiently metabolize resveratrol and to a lesser extent δ-viniferin; however, stilbene metabolization products were not characterized [41,42]. In this study, we investigated the diversity of secreted proteins of *N. parvum* and *D. seriata* cultured in media with and without grapevine wood by an MS-based proteomic analysis. Then, we focused on the detailed fate of different grapevine stilbenes (*trans*-resveratrol, *trans*-piceid, *trans*-piceatannol, and *trans*-δ-viniferin) after metabolization by extracellular *Botryosphaeriaceae* enzymes. Kinetics of stilbene metabolization were analyzed by LC-MS monitoring in correlation with laccase and Mn peroxidase enzyme activities. We were able to show that the two studied fungi had specific protein secretion profiles and that these profiles changed when they were grown in the presence of grapevine sawdust. Furthermore, we have also shown that both fungi were able to metabolize stilbenes more or less efficiently.

## 2. Materials and Methods

### 2.1. Fungal Material

*Botryosphaeriaceae* strains used in this study were already used and described in other publications [22,43,44]. *D. seriata* F98-1 and *N. parvum* Bourgogne S-116 were obtained from the collection of the IFV (Institut Français de la Vigne et du Vin, Rodilhan, France). *D. seriata* F98-1 was isolated in 1998 in Perpignan as described in Larignon et al. [6]. *N. parvum* Bourgogne S-116 was isolated in 2009 from Chardonnay plants showing decline in nurseries.

*D. seriata* 98.1 and *N. parvum* Bourgogne S116 have been characterized based on morphological aspect (mycelia growth and aspect, pycnidia production, size and shape of ascospores and conidia) as described by van Niekerk et al. [42]. The genome of *D. seriata* 98.1 has been sequenced (NCBI GenBank accession number MSZU00000000) [43].

Fungi were grown in Petri dishes containing PDA (Potato Dextrose Agar) solid medium at 27 °C in the dark. The fungi were subcultured every 10 days.

### 2.2. Extracellular Protein Extraction

Fungi were grown in solid cultures for 10 days; then, the mycelia of each strain (half of a petri dish) was introduced in a 500 mL Erlenmeyer flask containing 250 mL of liquid culture medium. Two different liquid culture media were used: 20 g/L malt medium and 20 g/L malt medium supplemented with 2.5 g of *Vitis vinifera* cv. Gewurztraminer sawdust. Liquid cultures were grown at 220× rpm in the dark at 27 °C during 21 days. After 21 days, three successive filtrations were carried out on 0.8, 0.45, and 0.2 µm on regenerated cellulose membranes (ReliaDisc, Ahlstrom, Finland).

According to Bénard-Gellon et al. [22], extracellular proteins were precipitated by the addition of ammonium sulfate (60% *w*/*v*) during 2 h at 4 °C under vigorous agitation. After centrifugation (10,000× rpm, 4 °C, 30 min), the pellet was collected, resuspended in deionized water, and dialyzed in 3.5 kDa cutoff tubing against deionized water for 20 h at 4 °C. The protein mixture was freeze-dried and stored at −20 °C for subsequent experiments.

For enzymatic activity measurements and kinetic monitoring of stilbene metabolization, freeze-dried proteins were solubilized in ultra-pure water at 2 mg/mL. Protein concentration was measured with a Nanodrop 1000 spectrophotometer (Thermo Scientific, Waltham, MA, USA) at 280 nm and adjusted at the appropriate concentration depending on subsequent experiments.

### 2.3. Enzymatic Activity of Wood Degradation

Laccase activity was determined with the ABTS (2,2′-azino-bis (3-ethylbenzothiazoline-6-sulfonic acid)) substrate. ABTS oxidation by laccase will produce the radical cation ABTS^•+^, producing a blue color that can be measured by UV spectrophotometer at 420 nm. In a 96-well plate (SpectraPlate^TM^-96 MB, PerkinElmer, Waltham, MA, USA), 50 µL of secreted proteins (2 mg/mL) were mixed with 50 µL of a solution containing 25 µL ABTS (2 mM in deionized water) and 25 µL sodium acetate buffer (100 mM, pH 4.5). The plates were incubated directly in the spectrophotometer (SpectraMax^®^ iD3, Molecular devices, San José, CA, USA) at 25 °C for 1 h. Then, the absorbance was read at 420 nm every 5 min after orbital shaking of 10 s. The absorbances obtained were converted into enzymatic activity (nmol/h/mg protein) using Beer–Lambert law (A=ε.l.c  → $c=\frac{A}{\varepsilon .l}$) with ε (36,000 M^−1^·cm^−1^) according to Mathieu et al. [44].

Manganese peroxidase activity was determined according to the procedure adapted from Mathieu et al. [44]. This method is based on the oxidative coupling of 3-methyl-2-benzothiazolinone (MBTH) and dimethylaminobenzene (DMAB), which was catalyzed by peroxidases in the presence of hydrogen peroxide (H_2_O_2_) and Mn^2+^, which releases indamine (purple) determined by spectrophotometry at 590 nm. A differential reaction containing 2 steps was carried out. The first step consists of measuring the activity of peroxidases in the presence of manganese and the second one consists of measuring the activity of peroxidase without manganese. The reaction in the presence of Mn^2+^ allows the determination of the Mn peroxidase activity. Two solutions, A and B, were used. Solution A was composed of a mixture of aqueous solutions: 5 mL of an equimolar mixture of sodium lactate/sodium succinate (100 mM each at pH 4.5), 0.5 mL of DMAB (50 mM), 0.5 mL of MBTH (1 mM), and 1 mL of manganese sulfate tetrahydrate (MnSO_4_-4H_2_O, 1 mM) for a final volume of 7 mL Solution B consisted of the same composition, except that MnSO_4_-4H_2_O was replaced by ethylenediaminetetraacetate (EDTA, 2 mM). For the analysis, 140 µL of solution A or B were mixed with 10 µL of H_2_O_2_ (1 mM) and 50 µL of secreted proteins (2 mg/mL) and incubated 1 h at 26 °C in a 96-well microplate (SpectraPlate^TM^-96 MB, PerkinElmer, Waltham, MA, USA). The absorbance was read at 590 nm every 5 min after orbital shaking of 10 s. The absorbance obtained with solution B was subtracted to the one obtained with solution A, which contained MnSO_4_-4H_2_O necessary to measure Mn peroxidase activity. The absorption values obtained were converted into enzymatic activity (nmol/h/mg protein) using Beer–Lambert law (A=ε.l.c  → $c=\frac{A}{\varepsilon .l}$) with ε (32,000 M^−1^·cm^−1^).

For both laccase and manganese peroxidase activity, a Wilcoxon test (*p* < 0.05) was performed using R studio software (R Development Core Team, 2010). Data were drawn using Prism 8 (v. 8.2.1). Error bars on histograms represent the standard error of the mean [45].

### 2.4. MS-Based Proteomic Analysis

#### 2.4.1. Sample Preparation

Freeze-dried proteins were resuspended in Laemli buffer, and about 25 µg were deposited and concentrated in a stacking gel. Gel bands were reduced with dithiothreitol (10 mM, 60 °C, 1 h) and alkylated with iodoacetamide (55 mM, RT, 20 min). After two dehydrations with acetonitrile (ACN), the proteins were cleaved with a modified porcine trypsin (Promega, Madison, WI, USA) solution at a 1:50 (*w*/*w*) enzyme: protein ratio. Digestion was performed overnight at 37 °C. Tryptic peptides were extracted using 60% ACN in 0.1% formic acid (FA). The excess of ACN was vacuum dried, and the samples were re-solubilized in 0.1% FA in water before nanoLC-MS/MS analysis.

#### 2.4.2. NanoLC-MS/MS Analysis

The nanoLC-MS/MS analysis was performed on a nanoACQUITY Ultra-Performance-LC (Waters Corporation, Milford, MA, USA) coupled to a Q-Exactive Plus mass spectrometer (Thermo Fisher Scientific, Waltham, MA, USA). Peptides were trapped on a Symmetry C18 pre-column (C18, 180 µm × 20 mm, 5 µm particle size, Waters), and separation was performed on an ACQUITY UPLC BEH130 C18 column (250 mm × 75 μm with 1.7 μm diameter particles). The solvent system consisted of 0.1% FA in water (solvent A) and 0.1% FA in ACN (solvent B). Samples were loaded into the pre-column over 3 min at 5 μL/min with 99% of A and 1% B. The peptides were eluted at 60 °C at a flow rate of 450 nL/min with the gradient of solvent B from 1 to 35% over 80 min.

The mass spectrometer was operated in positive mode, with the following settings: spray voltage 1800 V and capillary temperature 250 °C. The system was operated in Data-Dependent Acquisition mode with automatic switching between MS (mass range 300–1800 *m/z* with R = 140,000, automatic gain control (AGC) fixed at 3 × 10^6^ ions and a maximum injection time set at 50 ms) and MS/MS (mass range 200–2000 *m/z* with R = 17,500, AGC fixed at 1 × 10^5^ and the maximal injection time set to 100 ms) modes. The ten most abundant peptides were selected on each MS spectrum for further isolation and higher energy collision dissociation fragmentation, excluding unassigned and monocharged ions. The dynamic exclusion time was set to 60 s. The normalized collision energy (NCE) was fixed at 27 V. The complete system was fully controlled by Thermo Scientific™ Xcalibur™ software.

#### 2.4.3. Protein Identification

Mass data collected during nanoLC-MS/MS analyses were processed, converted into mgf peak list format with MSConvert, and interpreted using Mascot algorithm (v. 2.6.2, Matrix Science, London, UK) running on a local server. Searches were performed with an in-house generated protein database composed of protein sequences of *Botryosphaeriales* and *Vitis vinifera* (UniprotKB, release 2019). All entries belonging to the Taxonomy ID 45131 and 29760 were extracted, and common contaminants and reverse copies of all sequences were added thanks to the database toolbox of MSDA (https://msda.unistra.fr, [46]). The following parameters were used: (i) Trypsin/P was selected as enzyme, (ii) one missed cleavage was allowed, (iii) methionine oxidation and acetylation of the protein N-term were set as variable modifications and the carbamidomethylation of cysteine was set as a fixed modification, (iv) mass tolerance for precursor ions was set at 5 ppm and at 0.05 Da for fragment ions. Mascot results were loaded into the Proline software (Proline Studio Release 2.0, http://proline.profiproteomics.fr/ [47]). For validating PSM (Peptide Spectrum Matches), the following parameters were applied: a rank of 1, a minimal score of 25, and a minimal length of 7 amino acids. In addition, a maximum false discovery rate of 1% was applied to the protein set. A total proteins circular diagram was drawn using prism 8 (v. 8.2.1) and a Venn diagram was drawn using the jvenn online platform [48].

### 2.5. Kinetic Monitoring of Stilbene Metabolization

#### 2.5.1. Chemicals

*Trans*-resveratrol was purchased from Alfa Aesar (Kandel, Germany), *trans*-piceatannol was purchased from Extrasynthese (Genay, France), lyophilized horseradish peroxidase (HRP), hydrogen peroxide (30%), and *trans*-piceid were purchased from Sigma-Aldrich (St Quentin Fallavier, France). Other stilbenes were synthetized according to the procedure inspired by Li et al. [49] for which we used general experimental procedures: Thin Layer Chromatography (TLC) was performed on Merck 60 F254 silica gel. Silica gel 73–230 mesh (Merck, Darmstadt, Germany) was used for column chromatography. High-resolution mass spectrometry negative electrospray ionization (HRMS-ESI) was performed with Agilent 6510 accurate-mass Quadrupole-Time of Flight (Q-TOF). FTIR spectra were recorded by the ATR technique using a Perkin Elmer Spectrum Two N™. NMR spectra were recorded at 300 K in deuterated acetone on a Bruker Avance 400 MHz spectrometer. Chemicals shifts (δ) are indicated in ppm and coupling constants (*J*) are indicated in Hz.

Stilbene synthesis: a solution of HRP (1 mg/mL) in a phosphate buffer (20 mM, pH 8) was added to a solution of *trans*-resveratrol (1.32 mmol, 300 mg) in 1:1 *v*/*v* acetone/phosphate buffer 20 mM pH 8 (10 mL/mmol). The mixture was stirred at 40 °C and 271 µL of 30% H_2_O_2_ were added during 40 min (20 µL every 3 min). Then, the reaction mixture was extracted with ethyl acetate. The organic layer was washed with deionized water, dried over anhydrous magnesium sulfate, and concentrated under vacuum. The crude mixture was purified on silica gel column and eluted with a mixture of dichloromethane:methanol 9:1 (1% *v*/*v* NET3). Three fractions were collected and analyzed by HPLC-MS. Then, the fraction 2 (45 mg) was repurified on silica gel column and eluted with diethylether: ethylacetate 95:5 with few drops of THF. Then, *trans*-δ-viniferin and pallidol were obtained as a yellow amorphous powder (10 mg (3% yield) and 20 mg (7% yield), respectively). Leachianol F and leachianol G were obtained as impure mixture.

*Trans*-δ-viniferin: HRESI MS (−) *m/z* 453.1320 [M−H]^−^ (calcd for C_28_H_21_O_6_^−^ 453.1344) 499.1389 [M+HCOOH-H]^−^ (calcd for C_29_H_23_O_8_^-^ 499.1398); FTIR (ATR) ν_max_ cm^−1^: 3308, 1596, 1487, 1341, 1234, 1149; ^1^H NMR (400 MHz, acetone-d6) δ = 8.55 (1 H, br. s), 8.29 (1 H, s), 8.26 (1 H, s), 7.43 (1 H, dd, *J* = 1.8, 8.4 Hz), 7.24 (3 H, m), 7.06 (1 H, d, *J* = 16.4 Hz), 6.88 (4 H, m), 6.53 (2 H, d *J* = 2.0 Hz), 6.28 (1 H, t, *J* = 2.3 Hz), 6.25 (1 H, t, *J* = 2.0 Hz), 6.198 (2 H, d, *J* = 2.3 Hz), 5.45 (1 H, d, *J* = 8.0 Hz), 4.46 (1 H, d, *J* = 8.0 Hz); ^13^C NMR (100 MHz, acetone-d6) δ = 160.7, 159.9, 159.7, 158.6, 145.3, 140.8, 132.6, 132.3, 131.8, 129.2, 128.7, 128.7, 127.3, 124.0, 116.3, 110.2, 107.5, 105.8, 102.8, 102.5, 94.1, 57.9.

Pallidol: HRESI MS (−) *m/z* 453.1337 [M−H]^−^ (calcd for C_28_H_21_O_6_ 453.1338) 499.1315 [M+HCOOH-H]^−^ (calcd for C_29_H_22_O_8_ 498.1315); FTIR (ATR) ν_max_ cm^−1^: 3323, 1601, 1511, 1464, 1344, 1173, 1129, 836; ^1^H NMR (400 MHz, acetone-d6) δ = 7.6–8.4 (6 H, br), 6.98 (4 H, d, *J* = 8.6 Hz), 6.70 (4 H, d, *J* = 8.6 Hz), 6.62 (2 H, d, *J* = 1.8 Hz), 6.19 (2 H, d, *J* = 1.9 Hz), 4.57 (2H, s), 3.81 (2 H, s); ^13^H NMR (100 MHz, acetone-d6) δ = 159.41, 156.41, 155.40, 150.40, 137.82, 129.12, 123;33, 115.89, 103.42, 102.56, 60.58, 54.04. Spectral data are in agreement with the literature [49,50].

Mixture of Leachianol F and G. Data of leachianol F: HRESI MS (−) *m/z* 471.1439 [M-H]^−^ (calcd for C_28_H_23_O_7_^−^ 471.1449) 517.1497 [M+HCOOH-H]^−^ (calcd for C_29_H_25_O_9_^−^ 517.1504); ^1^H NMR (500 MHz, acetone-d6) characteristic signals δ = 6.86 (2 H, d, *J* = 8.2 Hz), 6.84 (2 H, d, *J* = 8.4 Hz), 6.72 (2 H, m), 6.67 (2 H, d, *J* = 8.7 Hz), 6.57 (1 H, d, *J* = 2.0 Hz), 6.30 (1 H, d, *J* = 2.0 Hz), 6.11 (1 H, t, *J* = 2.2 Hz), 5.91 (2 H, d, *J* = 2.1 Hz), 4.45 (1 H, dd, *J* = 7.8 and 4.8 Hz), 4.22 (1 H, d, *J* = 3.5 Hz), 4.02 (1 H, m) 3.35 (1 H, dd, *J* = 7.8 and 4.0 Hz). Data of leachianol G: HRESI MS (−) *m/z* 471.1439 [M−H]^−^ (calcd for C_28_H_23_O_7_^-^ 471.1449) 517.1497 [M+HCOOH-H]^−^ (calcd for C_29_H_25_O_9_^−^ 517.1504); ^1^H NMR (500 MHz, acetone-d6) main characteristic signals δ = 7.06 (2 H, d, *J* = 8.7 Hz), 6.86 (2 H, m), 6.21 (1 H, d, *J* = 2.0 Hz), 6.15 (1 H, t, *J* = 2.3 Hz), 6.14 (2 H, d, *J* = 2.0 Hz), 5.72 (1 H, d, *J* = 1.7 Hz), 4.26 (1 H, d, *J* = 3.5 Hz). Spectral data are in agreement with the literature [49,51].

#### 2.5.2. Preparation of Solutions

The kinetic monitoring was performed on four stilbenes: *trans*-resveratrol, *trans*-piceid, *trans*-piceatannol, and *trans*-δ-viniferin. For this purpose, we placed in a brown vial 50 µL of mixture of extracellular proteins of *D. seriata* (2 mg/mL) in contact with 5 µL of each stilbene at 0.5 mg/mL (in DMSO/MeOH 1:1) and 445 µL of sodium acetate buffer (20 mM, pH 4.5). In the case of *N. parvum*, an extracellular protein mixture at 2 mg/mL was added to 250 µL of a stilbene solution at 10 µg/mL (prepared from 20 µL of stilbenes at 0.5 mg/mL mixed with 980 µL of sodium acetate buffer). Thus, the final concentration of stilbenes was 5 µg/mL, and that of protein mixtures of *D. seriata* and *N. parvum* was 200 µg/mL and 1 mg/mL, respectively.

An external calibration of mixed stilbenes was realized to quantify the parent and metabolization products. Then, 20 µL of each stilbene at 0.5 mg/mL were mixed with 900 µL of sodium acetate buffer. From this stock solution, 4 daughter solutions were prepared at the following concentrations: 0.2, 0.5, 2.5, and 5 µg/mL. The amount of stilbene at T0 (before the contact between enzyme and stilbene) was measured by LC-MS analysis of a solution of each stilbene at 5 µg/mL in sodium acetate buffer prior to each stilbene metabolization monitoring by LC-MS with *D. seriata* and *N. parvum* protein mixtures.

Analyses were performed at a rate of one injection of 3 µL every 45 min at room temperature. Analyses were conducted over a maximum of 10.5 h for *D. seriata* proteins and for a maximum of 50 h for *N. parvum*.

Moreover, we analyzed *trans*-resveratrol metabolization by *Aspergillus* laccases and horse radish peroxidase (HRP) both purchased from Sigma Aldrich (St Quentin Fallavier, France). For this purpose, we mixed 50 µL of commercial *Aspergillus* laccase diluted at 1/100 in contact with 5 µL of *trans-*resveratrol at 0.5 mg/mL (in DMSO/MeOH 1:1) and 445 µL of sodium acetate buffer (20 mM, pH 4.5). In the case of HRP, 230 µL of enzyme solution at 0.01 mg/mL in sodium acetate buffer and 20 µL of 30% aqueous solution of H_2_O_2_ were added to 250 µL of a *trans*-resveratrol solution of at 10 µg/mL. We also performed the metabolization of *trans*-resveratrol by *Aspergillus* laccase with H_2_O_2_ and HRP without H_2_O_2_.

#### 2.5.3. HPLC-MS Analysis

The analytical system used was a High-Performance Liquid Chromatography Agilent 1100 series equipped with a DAD and coupled to an Agilent 6510 accurate-mass Quadrupole-Time of Flight (Q-TOF) mass spectrometer with electrospray ionization (ESI) source in negative ionization mode (Agilent Technologies, Santa Clara, CA, USA). Stilbenes were separated in a Zorbax SB-C18 column (3.1 × 150 mm, Ø 3.5 µm), equipped with a Zorbax Eclipse plus C18 pre-column (2.1 × 12.5 mm, Ø 5 µm, Agilent Technologies, Santa Clara, CA, USA). The mobile phase solvents were composed of 0.1% formic acid in LC-MS grade water (solvent A) and 0.1% formic acid in LC-MS grade methanol (solvent B), used in a gradient (A:B): 95:5 (0–3 min), 95:5 to 0:100 (3–23 min), 0:100 (23–33 min), and 95:5 (33–40 min). The flow rate was 0.35 mL/min. The injection volume of each sample was 2 µL. The drying gas flow and the nebulizer pressure were set at 13.0 L/min at 325 °C and at 35 psi, respectively. Other MS conditions included fragmentor: 150 V, capillary voltage: −3500 V, and collision energy: 20 V. Negative mass tune was performed with standard mix G1969-85000 (Agilent Technologies, Santa Clara, CA, USA).

The data acquisition system software was Agilent MassHunter v. B.02.00. Data processing was performed with Agilent MassHunter Qualitative and Quantitative software v. B.07.00. Absolute stilbene contents were obtained thanks to external calibration curves prepared with pure standards. Histograms and kinetics representation were drawn using prism 8 (v. 8.2.1). Error bars on histograms represent the standard error of the mean [45].

Stilbene quantification was performed according to the quantity of each specific stilbene used at the beginning of the experiments (T0). Stilbene concentrations at time X (Tx) were obtained by external calibration and reported as percentage of stilbene content at T0.

Concerning the piceid metabolization, the percentage of remaining piceid and the percentage of each metabolization products formed were calculated according to the following formulas, respectively: $\frac{{\left[\mathrm{piceid}\right]}_{\mathrm{Tx}}}{[{\mathrm{piceid}}_{T0}}\times 100$ and $\frac{{\left[\mathrm{MPy}\;\right]}_{\mathrm{TX}}}{{\left[\mathrm{piceid}\right]}_{T0}\times \mathrm{MW}\left(\mathrm{MPy}\right)/\left(2\times 390\right)}\times 100$, where [piceid]_Tx_ and [MPy]_Tx_ designed respectively the concentration of piceid metabolization product y at Tx obtained thanks to the external calibration, and MW(MPy) corresponds to the molecular weight of metabolization product y. The same corresponding formulas were used in the case of resveratrol metabolization $\left(\frac{{\left[\mathrm{resveratrol}\right]}_{\mathrm{Tx}}}{{\left[\mathrm{resveratrol}\right]}_{T0}}\times 100\;\mathrm{and}\;\frac{{\left[\mathrm{MPy}\;\right]}_{\mathrm{TX}}}{{\left[\mathrm{resveratrol}\right]}_{T0}\times \mathrm{MW}\left(\mathrm{MPY}\right)/\left(2\times 228\right)}\times 100\;\right)$ and piceatannol metabolization $\left(\frac{{\left[\mathrm{piceatannol}\right]}_{\mathrm{Tx}}}{{\left[\mathrm{piceatannol}\right]}_{T0}}\times 100\right)$.

## 3. Results

### 3.1. Extracellular Laccase and Mn Peroxidase Activities of N. parvum and D. seriata

It is well assumed that lignin degradation is achieved by synergistic enzyme activities such as lignin peroxidases, laccases, and manganese peroxidases. These activities could also be involved in stilbene degradation. In addition, we already reported that laccase and Mn peroxidase activities could be measured in extracellular protein extracts of *N. parvum* and *D. seriata* [41].

Since the extracellular enzyme activities could vary between fungal culture batches, we first checked the extracellular wood-degrading enzyme activities of the extracts used in this study (Figure S1). As previously described, we observed that laccase activity was enhanced by the addition of grapevine sawdust for both fungi. However, laccase activity was significantly higher for proteins secreted by *D. seriata* compared to proteins secreted by *N. parvum* (Figure S1a). As observed for laccase activity, Mn peroxidase activity was significantly higher for proteins secreted by *D. seriata* and mostly when cultivated with grapevine sawdust. In contrast to laccase activity, the addition of grapevine sawdust had no effect on Mn peroxidase activity for *N. parvum* (Figure S1b). These results are consistent with those from Stempien et al. and show that the enzymatic extracts could be used for further study of stilbene metabolization [41].

### 3.2. N. parvum and D. seriata Extracellular Protein Content Investigation

**Figure 1:**
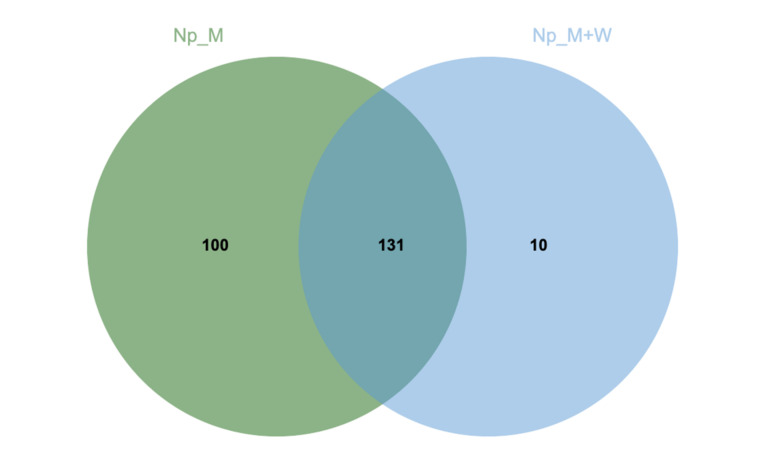
Venn diagram of *N. parvum* secreted proteins in malt medium (green) and in malt medium supplemented with grapevine sawdust (blue) adapted from [48]. Secreted proteins were extracted from two replicates.

In order to understand how *N. parvum* and *D. seriata* bypass the stilbene’s plant defense metabolism, proteomic analysis was conducted on extracellular proteins of fungi cultivated with or without grapevine sawdust. In total for *N. parvum* common across two replicates, we putatively annotated 231 secreted proteins divided into eight enzymatic classes such as hydrolases, isomerases, ligases, lyases, oxidoreductases, transferases, non-enzymatic proteins, and uncharacterized proteins. Among them, 131 identical proteins were found in both culture media, 100 were specifically secreted in malt medium, and 10 were only identified in malt medium supplemented with wood (Figure 1, Table S1).

**Figure 2:**
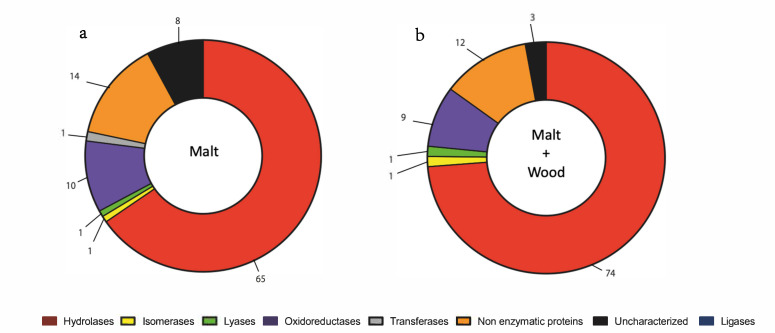
Classification of secreted protein based on molecular functions of *N. parvum* cultivated without (**a**) or with grapevine sawdust (**b**). The numbers are expressed in percentage of total proteins in each condition.

The target classes of enzymes identified were hydrolases, isomerases, lyases, oxidoreductases, transferases, non-enzymatic proteins, and uncharacterized (Figure 2a, Table S2). When *N. parvum* was cultivated with grapevine sawdust, the major protein family was hydrolases (74%) followed by oxidoreductases (9%) (Figure 2b). We observed a similar composition of extracellular enzymes with a higher proportion of hydrolases when *N. parvum* was cultivated with grapevine sawdust (74% with sawdust and 65% without) (Figure 2, Table S2).

**Figure 3:**
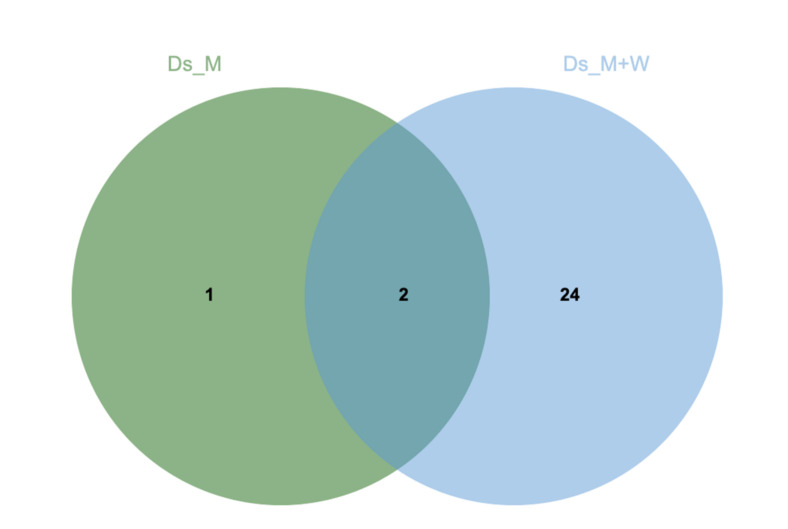
Venn diagram of *D. seriata* secreted proteins in malt medium (green) and in malt medium supplemented with grapevine sawdust (blue) adapted from [48]. Secreted proteins were extracted from three replicates.

Among all the proteins, only two fungal proteins were identified in both culture media, one was specifically secreted in malt media, and 24 were specific to culture in malt medium supplemented with wood (Figure 3, Table S3).

**Figure 4:**
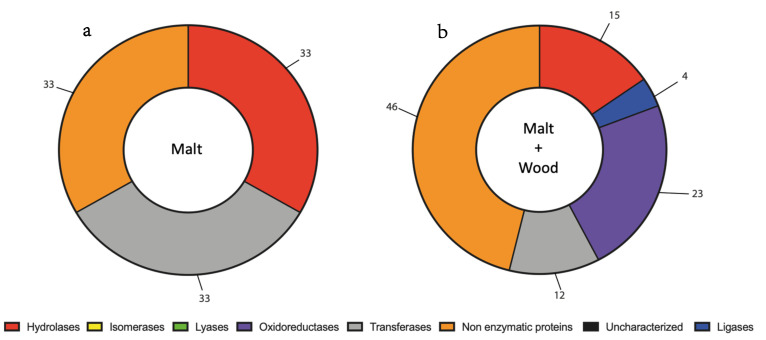
Classification of secreted protein based on molecular functions of *D. seriata* cultivated without (**a**) or with grapevine sawdust (**b**). The numbers are expressed in percentage of total proteins in each condition.

Concerning *D. seriata*, the target classes of enzymes identified were hydrolases, ligases, oxidoreductases, transferases, non-enzymatic proteins, and uncharacterized proteins (Figure 4, Table S4). When *D. seriata* was cultivated with grapevine sawdust, the major protein family was non-enzymatic proteins (46%) followed by oxidoreductases (23%) (Figure 4a, Table S4). The presence of oxidoreductases (23%) and ligases (4%) was only observed when *D. seriata* was cultivated with grapevine sawdust (Figure 4b, Table S4).

### 3.3. Kinetics of Stilbene Metabolization by Botryosphaeriaceae

In a previous study, we showed that *Botryosphaeriaceae* fungi are able to metabolize stilbenes, especially resveratrol [41]. However, metabolization products were not characterized. In this study, we analyzed precisely stilbene becoming in the presence of *Botryosphaeriaceae* extracellular proteins (from *N. parvum* and *D. seriata*). We focused our experiments on monomeric stilbenes and monitored the kinetics of each stilbene metabolization by LC-MS analysis.

First of all, preliminary experiments of kinetic monitoring with *trans*-resveratrol and extracellular proteins of *N. parvum* obtained from a fungal culture without grapevine sawdust did not show any metabolization reaction up to 24 h (data not shown). In contrast, the same monitoring with the same protein concentration obtained from a culture with grapevine sawdust showed a production of metabolization products from the first hour of contact. We also noticed that the amount of metabolization products formed was proportional to the quantity of proteins brought in the reaction. Therefore, the further experiments were carried out with stilbenes in the presence of extracellular proteins obtained from a fungal culture supplemented with vine sawdust. Kinetics were realized with total proteins at 1 mg/mL for *N. parvum* and 200 µg/mL for *D. seriata*. We used two distinct concentrations, since we expected a higher enzymatic activity for *D. seriata* proteins, as shown by Stempien et al. [41]. The kinetic monitoring was performed over 10.5 h for *D. seriata* and 50 h for *N. parvum*.

**Figure 5:**
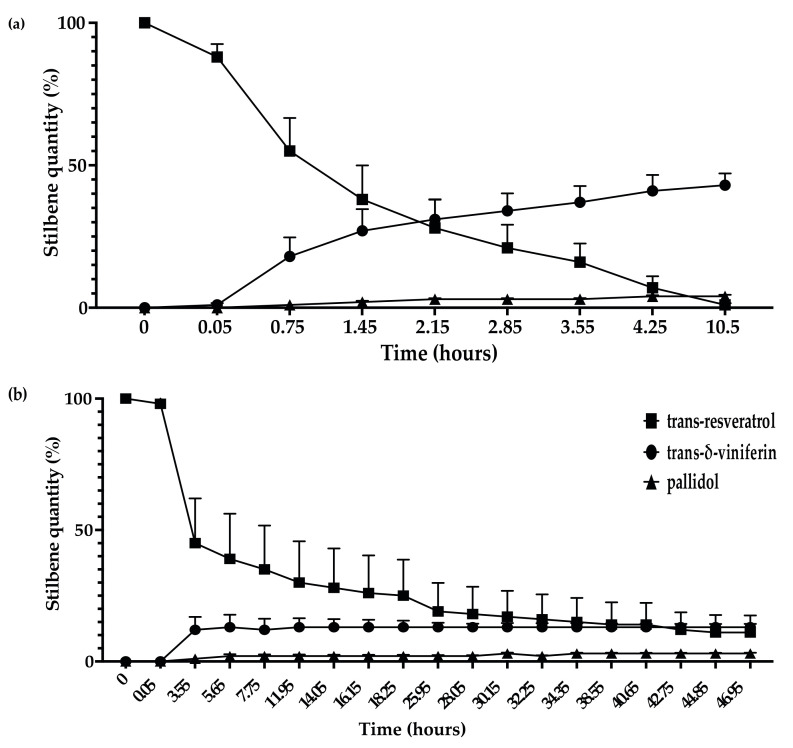
Kinetics of *trans*-resveratrol (5 μg/mL) metabolization in sodium acetate buffer by total extracellular protein extract from (**a**) *D. seriata* (200 μg/mL) and (**b**) *N. parvum* (1 mg/mL). Evolution of *trans*-resveratrol quantity is represented by square dots, *trans-δ*-viniferin is represented by circle dots, and pallidol is represented by triangle dots. Error bars represent the standard error of the mean (SEM). Mean and SEM were calculated with three biological replicates each comprising two technical replicates (*n* = 6).

The kinetics of resveratrol metabolization showed that the extracellular proteins of *D. seriata* were able to rapidly and efficiently metabolize *trans*-resveratrol compared to *N. parvum* (Figure 5a,b). Half (50%) of *trans*-resveratrol was metabolized between 0.75 and 1.45 h for *D. seriata*. The metabolization of *trans*-resveratrol was correlated with the increase in *trans*-δ-viniferin and pallidol contents. After 10.5 h, only 1% of *trans*-resveratrol was detected for 4% of pallidol and 43% of *trans-δ*-viniferin (Figure 5a). For *N. parvum*, we observed the same *trans*-resveratrol metabolization products but with a slower rate. Indeed, more than 50% of *trans*-resveratrol has been metabolized after 3.55 h. At the end of the experiment (46.95 h), we were able to detect 11% of *trans*-resveratrol, 3% of pallidol, and 13% of *trans-δ*-viniferin (Figure 5b).

**Figure 6:**
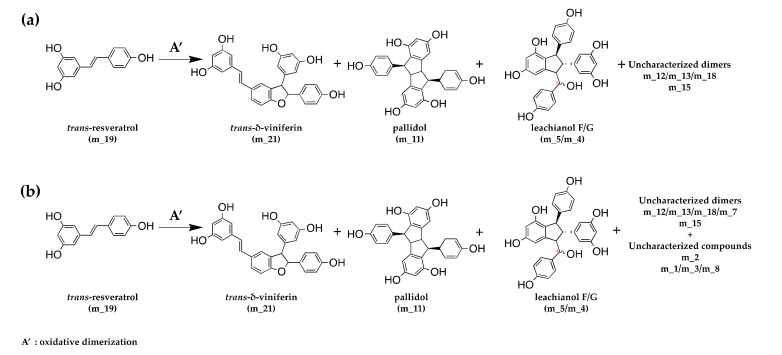
Schematic oxidative dimerization of *trans*-resveratrol by fungal extracellular proteins from (**a**) *D. seriata* and (**b**) *N. parvum*.

Concerning the LC-MS monitoring of *trans*-resveratrol (m_19) metabolization, we observed for both fungi the formation of *trans*-resveratrol dimers (Figure 6 and Table S5): two dimers of mass 454.14 (*trans-δ-*viniferin (m_21) and pallidol (m_11)) and five dimers *m/z* 472.15 (a mixture of leachianol F and G (m_5 and m_4) and three other dimers (m_12, m_13, and m_18)). These latter dimers consist of putative resveratrol dimers C_28_H_24_O_7_ that could correspond to restrytisol A and B and tricuspidatol A. We also observed one dimer *m/z* 486.17 (m_15), which consists of a C_29_H_26_O_7_ compound and is putatively identified as a methoxylated resveratrol dimer. In addition to these compounds, *trans*-resveratrol metabolization by *N. parvum* led to the formation of four additional compounds: one compound *m/z* 244.07 (m_1), two compounds *m/z* 244.18 (m_3 and m_8), one compound *m/z* 232 (m_2), and one *m/z* 472.15 (m_7) (Figure 6, Table S5).

**Figure 7:**
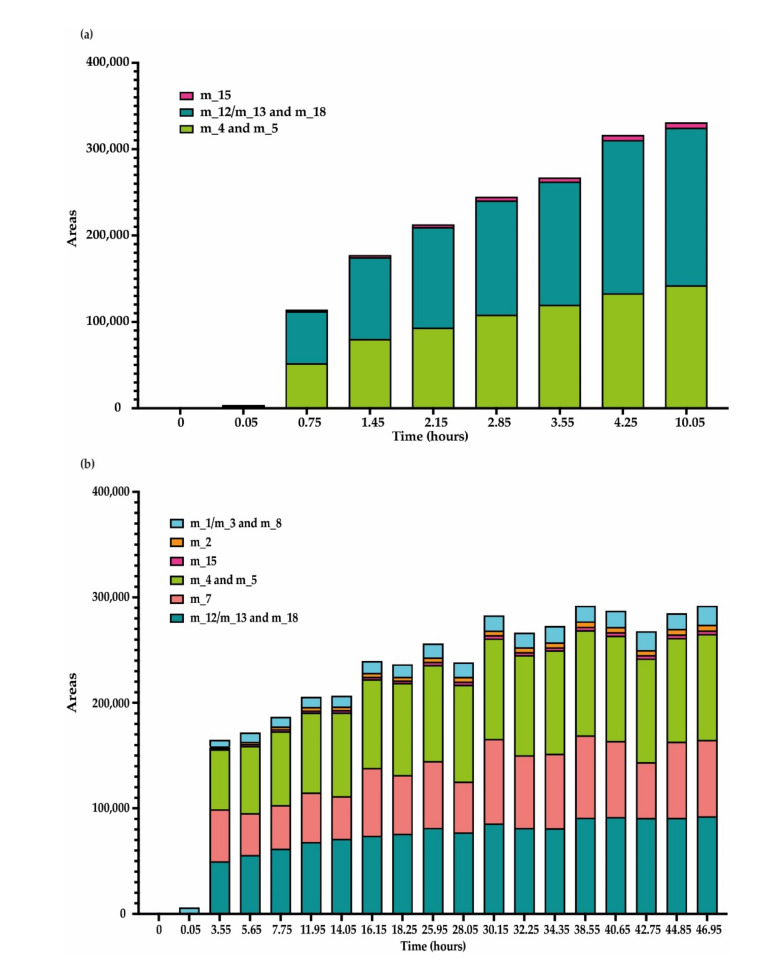
Putative metabolization products obtained during kinetic monitoring of *trans*-resveratrol (5 μg/mL) metabolization in sodium acetate buffer by total extracellular protein extract from (**a**) *D. seriata* (200 μg/mL) and (**b**) *N. parvum* (1 mg/mL). The mean was calculated over three biological replicates, each comprising two technical replicates (*n* = 6).

Putative compounds and leachianol F/G were not quantified because they could not be purified or obtained with sufficient purity, so only areas of these compounds were reported (Figure 7). We observed an increase in the relative abundance (areas) of these compounds concomitant to the decrease in *trans*-resveratrol content (Figure 7). For both fungi, the main unknown compounds formed after resveratrol metabolization are dimers m_4/5 and m_12/13. For *D. seriata* at the end of the analysis, we observed more compounds m_12/13 (area = 165,621) than m_4/5 (area = 142,250). The inverse observation was made for *N. parvum* that was able to synthesize more diverse resveratrol metabolization products, especially m_7 dimer, which was specific of this fungus. Other minor compounds were also specific to *N. parvum* proteins (m_1, m_8, m_3, and m_2).

**Figure 8:**
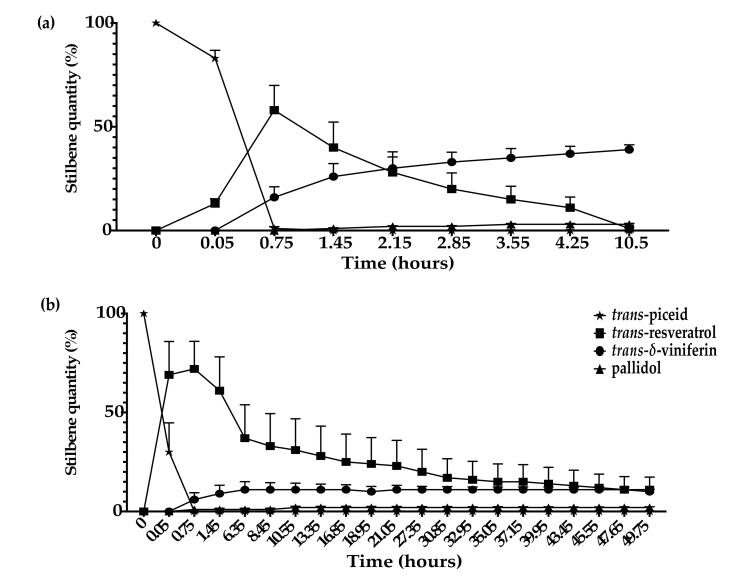
Kinetics of *trans*-piceid (5 μg/mL) metabolization in sodium acetate buffer by total extracellular protein extract from (**a**) *D. seriata* (200 μg/mL) and (**b**) *N. parvum* (1 mg/mL). Evolution of *trans*-piceid contents is represented by star dots, *trans*-resveratrol is represented by square dots, *trans*-δ-viniferin is represented by circle dots, and pallidol is represented by triangle dots. Error bars represent the standard error of the mean (SEM). Mean and SEM were calculated over three biological replicates each comprising two technical replicates (*n* = 6).

In addition, we observed that the extracellular proteins of *N. parvum* and *D. seriata* were able to efficiently metabolize *trans*-piceid. For *D. seriata*, complete metabolization of *trans*-piceid occurred within 0.75 h. *Trans*-piceid was first deglycosylated in *trans*-resveratrol, which represented 58% of the total metabolization mixture of *trans*-piceid. Shortly after resveratrol formation, we could observe the apparition of *trans-δ*-viniferin representing 16% of the compounds. It is likely that piceid is first deglycosylated in resveratrol, which in turn is oxidized to *trans*-δ-viniferin by extracellular proteins. After 1.45 h, the metabolization of 50% of *trans*-resveratrol was observed. After 10.5 h, only 1% of *trans*-resveratrol was detected for 3% of pallidol and 39% of *trans-*δ*-*viniferin (Figure 8a). The metabolization of *trans*-piceid was also performed for *N. parvum* but with a slower rate. The complete *trans*-piceid deglycosylation took place during the same time, i.e., 0.75 h. At that point, we observed 69% of *trans*-resveratrol even though the maximum (72%) was reached 0.7 h later. At the end of the experiment (49.75 h), we were able to detect 11% of *trans*-resveratrol, 10% of *trans-*δ-viniferin, and 2% of pallidol (Figure 8b).

**Figure 9:**
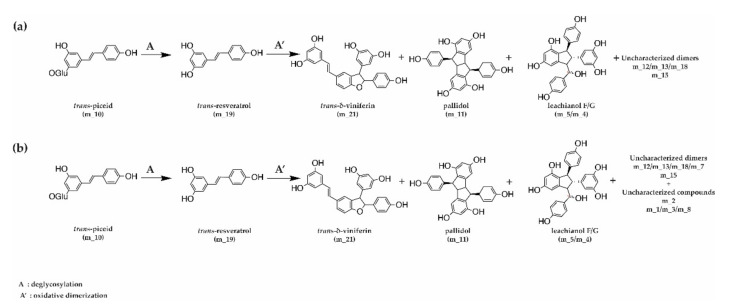
Schematic metabolization of *trans*-piceid by fungal extracellular proteins from (**a**) *D. seriata* and (**b**) *N. parvum*.

Concerning *trans*-piceid (m_10) metabolization, we observed in a first step its deglycosylation leading to the formation of *trans*-resveratrol. Before the complete disappearance of *trans*-piceid, dimer compounds started to be produced. These compounds are the same as those formed during *trans*-resveratrol metabolization and with the same distribution profile (Figure 9 and Figure S2). Regarding the relative abundance of compounds detected but not quantified, we observed after *trans*-resveratrol metabolization similar increases of these same compounds (Figure S2). Production of the dimers m_4/m_5, m_12/m_13, m_15, and m_18 appears to be more abundant in *N. parvum* compared to *D. seriata,* as already observed for *trans*-resveratrol metabolization.

**Figure 10:**
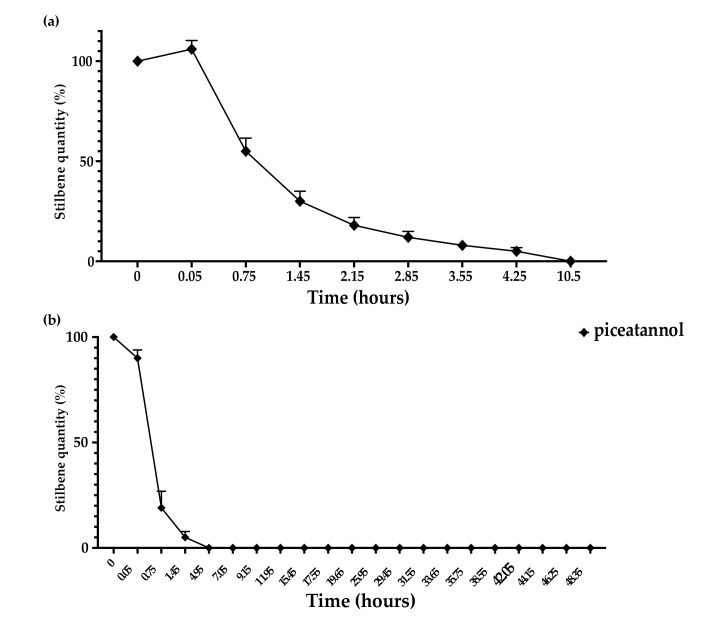
Kinetics of *trans*-piceatannol (5 μg/mL) metabolization in sodium acetate buffer by total extracellular protein extract isolated from (**a**) *D. seriata* (200 μg/mL) and (**b**) *N. parvum* (1 mg/mL). Evolution of *trans*-piceatannol contents is represented by diamond dots. Error bars represent standard error of the mean (SEM). Mean and SEM were calculated over three biological replicates, each comprising two technical replicates (*n* = 6).

Concerning *trans*-piceatannol, we observed that both extracellular proteins of *D. seriata* and *N. parvum* were able to metabolize this compound. The complete metabolization of *trans*-piceatannol by *D. seriata* was achieved in 10.5 h, while the full metabolization by *N. parvum* occurred in only 4.95 h (Figure 10a,b).

**Figure 11:**
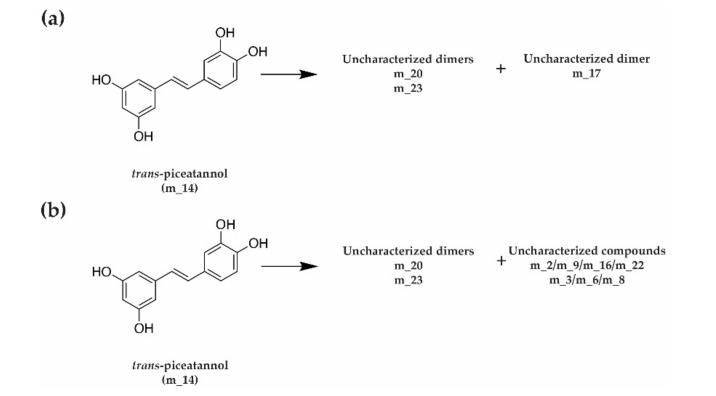
Schematic metabolization of *trans*-piceatannol by fungal extracellular proteins from (**a**) *D. seriata* and (**b**) *N. parvum*.

In the case of *trans*-piceatannol (m_14) metabolization, we noticed a different profile of metabolites between the two fungal species (Figure 11). For both fungi, we observed a production of *trans*-piceatannol dimers (m_20 (mass 486.13) and m_23 (484.12)). In addition, for *D. seriata*, we observed the formation of an additional dimer 486.13 (m_17), which was not present for *N. parvum*. Moreover, with *N. parvum* proteins, we detected the formation of several specific compounds: two compounds of mass 232.13 (m_2 and m_22), two compounds of mass 232.07 (m_9 and m_16), and three compounds of mass 244.18 (m_3, m_6, and m_8) (Figure 11 and Table S5). According to the mib-polyphenol database (PRD: Stilbenes, Viniferin, Resveratrol and derivatives, PRDb-ISVV) and based on the detected masses, the dimeric form m_23 (mass 484.12) might be cassigarol D or cassigarol G. According to the same database, dimeric compounds *m/z* 486 might be jezonodione, cararosinol D, cassigarol E, longusol C, maackin, maackin A, tibeticanol, scirpusin B (*cis* or *trans*), or gneafricanin C. We performed MS/MS analysis and dimer m_20 recorded for both fungi could be assigned to cassigarol E (Table S5).

**Figure 12:**
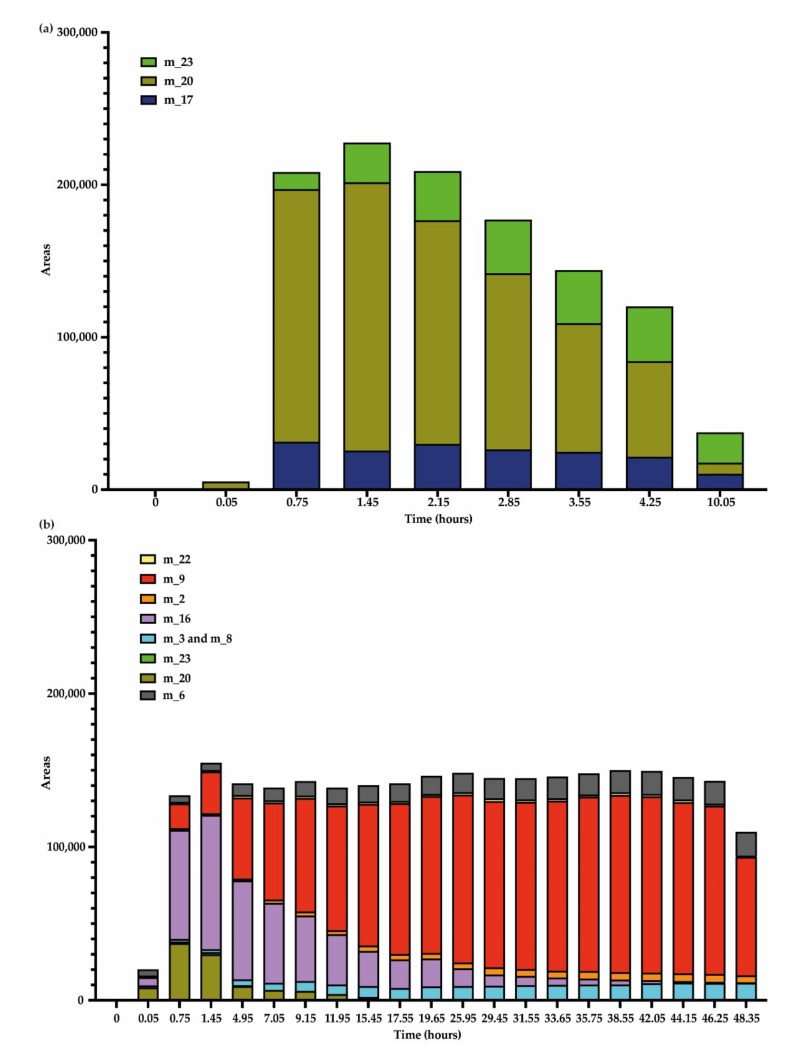
Unknown/Putative metabolization products observed during kinetic monitoring of *trans*-piceatannol (5 μg/mL) metabolization in sodium acetate buffer by total extracellular protein extract from (**a**) *D. seriata* (200 μg/mL) and (**b**) *N. parvum* (1 mg/mL). Mean was calculated over three biological replicates each comprising two technical replicates (*n* = 6).

For the non-quantified metabolites (Figure 12), we observed for *D. seriata* a transient increase of these compounds followed by a rapid metabolization. For the compound m_23, we observed a maximum area at 4.25 h, this compound decreasing thereafter. Compound m_20 was detected from the beginning of the experiment. We observed a maximum area at 0.75 h for m_17 and 1.45 h for m_20. For piceatannol metabolization with *N. parvum* proteins, we also detected compounds m_20 and m_23. The formation of these two compounds was also transient. In addition, we also observed the specific formation of compounds m_2, m_3, and m_8, which were already detected after resveratrol metabolization by *N. parvum*. These three compounds increased during the whole kinetic monitoring and were still detected at the end of the experiment. Compound m_6 followed the same trend. Regarding the compounds m_9 and m_16, we observed their formation followed by a decrease. Concerning compound m_22, it was present from the start of the analysis and varied very slightly with a decreasing trend.

Finally, we investigated *trans*-δ-viniferin metabolization by *D. seriata* and *N. parvum* extracellular proteins. The experiment was performed over 14 days, and there was no variation in *trans*-δ-viniferin content in control experiment (without protein). In the presence of proteins, we observed a more important decrease in *trans*-δ-viniferin during experiments with *D. seriata* (about 50% of control) compared to *N. parvum* proteins (>20%), but no metabolization products were detected (data not shown).

### 3.4. Kinetics of Stilbene Metabolization by Commercial Products

In order to precisely identify the enzymes responsible for stilbene metabolization, we tested the activity of different commercial enzymes in the presence of *trans*-resveratrol. To test the laccase activity, we used a laccase mixture from *Aspergillus*, and for the manganese peroxidase activity, we used a horseradish peroxidase (HRP). We performed the same kinetic monitoring analysis as for *Botryosphaeriaceae* proteins.

**Figure 13:**
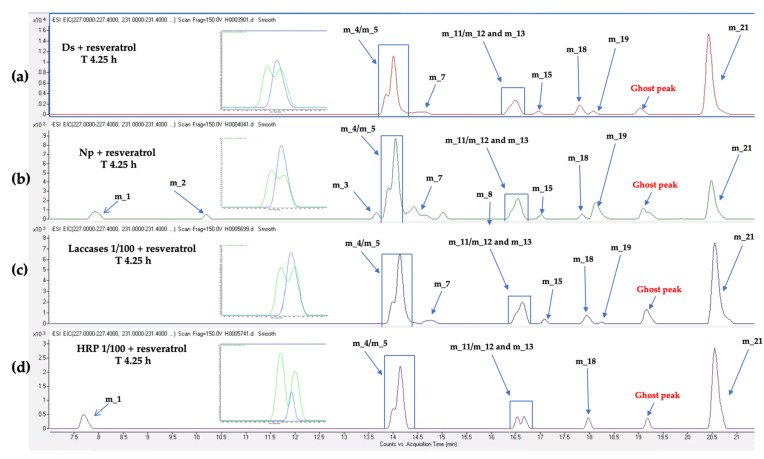
Extracted Ion Chromatogram (EIC) obtained during resveratrol metabolization (5 μg/mL) after 4.25 h by (**a**) *D. seriata* extracellular proteins (200 μg/mL), (**b**) *N. parvum* (1 mg/mL), (**c**) *Aspergillus* laccases diluted at 1/100 from a commercial solution (10 LAMU/g, LAMU: quantity of enzyme oxidizing 1 micromole of syringaldazine per minute at pH 7.5 and 30 °C), (**d**) HRP (0.01 mg/mL) + 5 μL H_2_O_2_. A zoom on m_12/m_13 and m_11 is presented on the left side of each chromatogram. The corresponding ion of pallidol (m_11) is highlighted in blue and that of m_12/m_13 is highlighted in green.

As shown in Figure 13, we could detect the formation of the same resveratrol dimers with the commercial laccase mixture from *Aspergillus* and with the total extracellular proteins from *D. seriata* and *N. parvum*. With commercial laccase, the stilbene profile was thus very similar to the one obtained after resveratrol metabolization with *N. parvum* and *D. seriata* proteins. In the case of resveratrol metabolization by HRP, it appears that compound *m/z* 486.17 (m_15) is not produced, and the pallidol proportion is lower (zoom on Figure 13). These results indicate that the metabolization recorded from the extracellular protein mixture of *Botryosphaeriaceae* might be initiated and realized by laccases. We also observed additional peaks for *N. parvum* total extracellular proteins compared to those present with laccases, HRP, or *D. seriata* extracellular proteins. This suggests that the recorded metabolization could be due to other enzymatic activities.

In parallel, we performed additional tests in the presence or absence of H_2_O_2_ to confirm a predominant laccase activity in the extracellular protein extract of the two *Botryosphaeriaceae*. No metabolization of *trans*-resveratrol was recorded when we used HRP without H_2_O_2_, attesting the necessity of this substrate for the peroxidase activity (data not shown). On the other hand, when H_2_O_2_ was added with laccases from *Aspergillus,* no more metabolization products were formed (data not shown). This experiment was also carried out with extracellular proteins of *D. seriata* and led to the same result (data not shown).

## 4. Discussion

In this study, we investigated the diversity of secreted proteins and extracellular enzyme activities involved in wood degradation and stilbene metabolization for *N. parvum* and *D. seriata*, which are two major fungi associated with Botryosphaeria dieback. To this aim, we studied their secreted proteins, their laccase and manganese peroxidase activities, as well as their ability to metabolize *trans-*resveratrol, *trans-*piceid, *trans-*piceatannol, and *trans-*δ-viniferin.

Concerning the activity of wood-degrading enzymes (laccases and manganese peroxidases), it is remarkable that the laccase activity of *D. seriata* is 6.5 times higher than that of *N. parvum*. For both fungi, we show that the addition of wood into the culture media triggered an increase in laccase activity. Even though the addition of wood induced laccase activity in *N. parvum*, it did not reach the level of the activity observed for *D. seriata*. Our study also shows that *D. seriata* is characterized by a manganese peroxidase activity 10 times higher than the one observed for *N. parvum* in the presence of wood. It is also interesting to note that the basal activity level of manganese peroxidase for both fungi in the absence of wood is very similar. Moreover, in contrast to *D. seriata*, the addition of wood did not enhance secreted peroxidase activity for *N. parvum*. Our results are consistent with those of Stempien et al., showing that *D. seriata* has stronger extracellular laccase and manganese peroxidase activities than *N. parvum* [41].

Regarding the analysis of proteins secreted by the two fungi, our study revealed that *N. parvum* secreted significantly more proteins than *D. seriata*. Indeed, whereas 231 different proteins were detected in the extracellular medium of *N. parvum*, only three were detected for *D. seriata* in the absence of wood supply. After wood addition, 141 proteins were detected for *N. parvum* and 29 were detected for *D. seriata*. These results are in agreement with the results observed by Bénard-Gellon et al. showing that *N. parvum* produced a higher quantity of total extracellular proteins compared to *D. seriata* [22]. In addition, Nagel et al. has shown a higher number of secreted proteins (CAZymes) in *N. parvum* compared to *D. seriata* through a genomic analysis of 26 *Botryosphaeriaceae* [52]. Our proteomic study also showed that in contrast to *D. seriata*, the addition of wood did not significantly enhance the number of secreted proteins for *N. parvum*. The majority of secreted enzymes of *N. parvum* were hydrolases, which represented 65% and 74% of the total proteins detected without and with grapevine sawdust, respectively. It is interesting to emphasize that *N. parvum*, which is known to be more aggressive than *D. seriata*, is characterized in the same volume by a higher amount and diversity of secreted proteins, especially hydrolases (including glycosidases) and oxidoreductases (including laccases and peroxidases) that are likely involved in cell wall and lignin degradation. In contrast, even if a lower production of secreted proteins was observed for *D. seriata*, the addition of grapevine wood significantly enhanced the extracellular protein production (three without wood powder compared to 29 with wood powder). This increase especially concerned proteins from the oxidoreductase class, which were only detected in the presence of grapevine wood. In this way, the profile of the secreted enzymes of *D. seriata* is consistent with the high inducibility of laccase and peroxidase activities by wood. In the presence of grapevine wood, a high number of secreted proteins also belong to the non-enzymatic proteins class for *D. seriata.*

Our proteomic study is consistent with previous work using genomic and transcriptomics studies to decipher the *Botryosphaeriaceae* secreted repertoire of virulence factors. Several studies have found a number of genes encoding secreted hydrolytic enzymes in the genome of *Botryosphaeriaceae*, including *N. parvum* and *D. seriata* [29,53,54]. Moreover, Quieroz and Santana proposed that fungi with a double cycle of life (latent and pathogenic) possess a large number of CAZymes involved in wood decay through the degradation of cellulose, hemicellulose, and pectin [53]. Morales-Cruz et al. also identified potential virulence factors in the genome of several fungi associated with GTDs. They showed an expansion of genes encoding lignin and cell wall degradation enzymes, as well as enzymes involved in toxin synthesis [27]. Similarly, Nagel et al. found that *Botryosphaeriaceae* had gene expansions of CAZymes, which are major constituents of the secretome. These genomic studies are in accordance with our proteomic analysis showing that most of the secreted *Botryosphaeriaceae* proteins belong to the hydrolase and oxidoreductase classes. Accordingly, Stempien et al. showed that *N. parvum* and *D. seriata* were able to degrade parietal polysaccharides via cellulase and hemicellulase activities. In addition, *N. parvum* has stronger cellulase and hemicellulase activities than *D. seriata*. This result is consistent with the higher number of hydrolases we found in secreted proteins of *N. parvum* compared to *D. seriata*.

In a recent study, Massonnet et al. (2018) reported that the change in growth substrate triggers major transcriptional reprogramming of virulence-associated genes in *N. parvum*. Indeed, when grapevine wood was added as a carbon source, a number of genes were upregulated compared to culture on PDA medium [54]. These differentially expressed genes were putative CAZymes, oxidoreductases, ligninases, and a large set of transporter genes. These results can be explained by the repression of genes encoding cell wall-degrading enzymes in filamentous fungi when preferred carbon sources (e.g., glucose) are present. Surprisingly, whereas wood supply enhanced the laccase activity in both *N. parvum* and *D. seriata*, and peroxidase activity in *D. seriata*, we did not detect a higher number of secreted hydrolases or oxidoreductases for *N. parvum* after wood supply. This result could be explained by the fact that we used an already rich medium (malt) supplemented with wood. In contrast, wood supply clearly enhanced the number of secreted proteins, especially oxidases in *D. seriata*.

Importantly, our work reveals that secreted *Botryosphaeriaceae* proteins produced after grapevine wood addition are able to rapidly metabolize the grapevine phytoalexin stilbenes. We show that *trans*-piceid was metabolized to resveratrol by a first deglycosylation step. Several authors have shown the bio-transformation of *trans*-piceid (also named polydatin) into *trans*-resveratrol by isolated enzymes (piceid-*O*-β-D-glucosidase) or whole microorganisms (*Aspergillus niger* alone or in addition with *Saccharomyces cerevisiae*) [55,56,57]. In our study, *trans*-resveratrol used directly or after *trans*-piceid deglycosylation is metabolized by oxidative dimerization and leads to different types of dimeric forms, the most abundant being *trans*-δ-viniferin, leachianol F/G, and the mixture of dimeric forms. For the same compounds, the increase appears to be lower in *N. parvum* compared to *D. seriata*. This can be explained by the fact that we observed a very low amount of *trans*-resveratrol at the end of the analysis (1%) for *D. seriata,* while we still observed 11% of the remaining *trans*-resveratrol for *N. parvum.* Overall, both *trans*-resveratrol metabolization and synthesis of biotransformation products appears to be faster in *D. seriata* compared to *N. parvum*. When we tested *trans*-resveratrol metabolization by commercial laccase or *D. seriata* proteins in the presence of H_2_O_2_, we did not observe any resveratrol transformation (data not shown). This suggests a possible inhibition of laccase by H_2_O_2_. Milton et al. indeed showed that the presence of H_2_O_2_ produced by glucose oxidase could inhibit the laccase activities [58]. Furthermore, no metabolization of *trans*-resveratrol was recorded when we used commercial HRP without H_2_O_2_, attesting the necessity of this substrate for the peroxidase activity. Since our studies were conducted in the absence of H_2_O_2_, stilbene metabolization is likely to be realized by fungal laccases. This is consistent with laccase activities in our experiments as well as with the high number of oxidases found among secreted *Botryosphaeriaceae* proteins and with the literature data for other fungi. Indeed, Cichewicz et al. has shown the dimerization of *trans-*resveratrol by *Botrytis cinerea* (*B. cinerea*) leading to leachianol F, restrytisols A, B, and C, pallidol, and *trans*-δ-viniferin production [59]. *trans*-δ-viniferin was also reported as a metabolization product of *trans-*resveratrol by *B. cinerea* by Breuil et al. [60]. Moreover, Pezet reported that *trans*-resveratrol dimerization was attributed to laccase-like oxidase secreted by fungi [61]. Beneveneti et al. also reported the oligomerization of phenolic compounds by purified laccases of *Trametes versicolor*, which is a white rot fungus [62], notably the transformation of *trans*-resveratrol into *trans*-δ-viniferin. Recently, using the secretome of *B. cinerea,* Gindro et al. have demonstrated the dimerization of *trans*-resveratrol into several compounds such as leachianol F/G, restrytisol B, *trans*/*cis*-δ-viniferin, and *3β-*(3′,5′-dihydroxyohenyl*)-2α*-(4″-hydroxyphenyl)dihydrobenzofuran-5-carbaldehyde [63].

In contrast to resveratrol, the metabolization of *trans*-piceatannol was faster with extracellular proteins from *N. parvum* than with proteins from *D. seriata*. To our knowledge, it is the first report of piceatannol metabolization by microorganisms or fungi extracellular enzymes. During the metabolization of piceatannol, we observed different profiles between the two fungi. Nevertheless, we observed two identical dimers (m_20 and m_23) produced by *N. parvum* and *D. seriata* enzymes. Finally, we noted dimers (m_1, m_3, and m_8) already found during the metabolization of resveratrol by *N. parvum*. Overall, a higher diversity of resveratrol and piceatannol metabolization products was found with enzymes of *N. parvum* compared to *D. seriata*. This could be related to a higher number and diversity of secreted proteins for this fungus, especially oxidases.

Several studies have reported an increase in the dimeric forms of stilbenes in organs of vines affected by trunk diseases. Stempien et al. showed an accumulation of *trans*-δ-viniferin after the treatment of suspension cells of *V. vinifera* cv. Gewurztraminer and *V. rupestris* with extracellular enzymes of *Botryosphaeriaceae* (*N. parvum* Bt67 and Bourgogne S-116 and *D. seriata*) [40]. Labois et al. also reported an increase in several dimeric forms including *trans*-δ-viniferin and pallidol in detached canes 7 days after inoculation with *N. parvum* Bt67. In addition, these authors showed an increase in the amount of *trans*-resveratrol and *trans*-piceatannol at 3 days after inoculation followed by a decrease at 7 days, which could be the result of the metabolization of these compounds into dimeric forms [38]. On the other hand, Calzarano et al. showed in grapevine leaves infected by grapevine leaf stripe disease that *trans*-resveratrol was the most abundant stilbene, and its dimerization was only partial. It is known that fungi involved in GTDs were not present in the leaves, so the higher resveratrol metabolization recorded in infected wood, compared to leaves, might be the result of plant response to the presence of fungi [34]. Nevertheless, according to Labois et al., we can hypothesize that the formation of dimers (such as pallidol, *trans*-δ-viniferin, leachianol F/G, and restritysol A) is achieved by the plant or by the fungus and that the other detected dimers found in the wood are exclusively from plant origin. Surprisingly, *N. parvum,* which is considered to be more aggressive than *D. seriata* [43,64], seems to have a slower ability to metabolize *trans*-resveratrol but a higher metabolization of *trans*-piceatannol. We could think that the detoxification of vine stilbenes by pathogens would be an avoidance mechanism to metabolize resveratrol into less toxic molecules. Since resveratrol is mainly transformed into δ-viniferin, this hypothesis is in contradiction with the fungistatic activity of this metabolite, which is significantly higher to that of *trans*-resveratrol [65]. Indeed, Khattab et al. recently showed a positive correlation between the rate and magnitude of resveratrol and *trans*-δ-viniferin accumulation and the resistance of different *Vitis vinifera* subsp. *sylvestris* accessions to *Botryosphaeriaceae* [65]. In our experiments, we did not observe *trans*-δ-viniferin metabolization by *Botryosphaeriaceae* extracellular proteins. It is possible that the metabolization of *trans*-δ-viniferin involves an internalization process and is realized by intracellular enzymes that we were not able to test here.

## 5. Conclusions

In conclusion, this is the first study to investigate the capacity and efficiency of stilbene metabolization by the extracellular enzymes of *D. seriata.* and *N. parvum*. The metabolization kinetics of stilbenes are faster with *D. seriata* and seem to be correlated with measured laccase activity and the higher proportion of secreted enzymes with oxidoreductase activity. To date, studies on resveratrol oligomers suggest that most of them would be endogenously produced in planta, although some may be formed via pathogenic fungal oxidases. Further analysis is needed to find out if resveratrol oligomerization is part of a fungal detoxification process or if it is part of an advanced plant beneficial defense mechanism using the cellular machinery of pathogens for their own benefit to eradicate them.

## Supplementary Materials

The following are available online at https://www.mdpi.com/article/10.3390/jof7070568/s1, Figure S1: Enzymatic activities: (a) Laccase activity, (b) Manganese peroxidase activity) measured for total secreted proteins (2 mg/mL) from *N. parvum* (black) and *D. seriata* (light gray). Protein extracts were isolated from culture filtrates of fungi grown in malt medium (Malt) and malt medium supplemented with grapevine wood (Malt + Wood). Activity is expressed in nmol/h/mg of protein. Means with * (*p* < 0.05), ** (*p* < 0.005), and *** (*p* < 0.0001) are significantly different (Wilcoxon test). Error bars represent standard error of the mean (SEM). Mean and SEM were calculated with three biological replicates each comprising two technical replicates (*n* = 6). Figure S2: Extract Ion Chromatograms (EICs) obtained from piceid metabolization by (a) *D. seriata* and (b) *N. parvum* extracellular proteins at different time points, Figure S3: Extract Ion Chromatograms (EICs) obtained from piceatannol metabolization by (a) *D. seriata* and (b) *N. parvum* extracellular proteins at different time points, Figure S4: Putative metabolization products obtained during kinetic monitoring of *trans*-piceid (5 μg/mL) metabolization in sodium acetate buffer by total extracellular protein extract from (a) *D. seriata* (200 μg/mL) and (b) *N. parvum* (1 mg/mL). Mean was calculated over three biological replicates, each comprising two technical replicates (*N* = 6), Table S1: *N. parvum* secreted proteins found according to the culture medium (malt, malt with wood, and both), Table S2: *D. seriata* secreted proteins found according to the culture medium (malt, malt with wood, and both), Table S3: List of *N. parvum* secreted proteins according to the culture medium, Table S4: List of *D. seriata* secreted proteins according to the culture medium, Table S5: List of compounds detected during *trans*-piceid, *trans*-resveratrol, and *trans*-piceatannol metabolization. ^a^ indicated metabolites with confirmed identification by standard, ^b^ indicated putative annotation by MS/MS and UV and ^c^ indicates non-characterized metabolites.
