# Supplementary material for: Comparative Study of Secreted Proteins, Enzymatic Activities of Wood Degradation and Stilbene Metabolization in Grapevine Botryosphaeria Dieback Fungi
Source: J Fungi (Basel). 2021 Jul 16;7(7):568. doi: 10.3390/jof7070568 (PMC8303417; doi:10.3390/jof7070568)
Supplement: Supplementary file 1 [file jof-07-00568-s001.zip › jof-1274657-supplementary.pdf]

Journal of *Fungi*

MDPI

Supplementary Material

Comparative Study of Secreted Proteins, Enzymatic Activities of Wood Degradation and Stilbene Metabolization in Grapevine Botryosphaeria Dieback Fungi

Clément Labois [1,2], Elodie Stempien [1,†], Justine Schneider [3,‡], Christine Schaeffer-Reiss [3], Christophe Bertsch [1], Mary-Lorène Goddard [1,2,*] and Julie Chong [1,*]

[1] Laboratoire Vigne, Biotechnologies et Environnement (LVBE, UPR 3991), Université de Haute Alsace, 68000 Colmar, France; clement.labois@uha.fr (C.L.); elodie.stempien@gmail.com (E.S.); christophe.bertsch@uha.fr (C.B.)

[2] Laboratoire d'Innovation Moléculaire et Applications, Université de Haute-Alsace, Université de Strasbourg, CNRS, LIMA, UMR 7042, CEDEX, 68093 Mulhouse, France

[3] Laboratoire de Spectrométrie de Masse BioOrganique (LSMBO), IPHC, Université de Strasbourg, CNRS, UMR7178, 25 Rue Becquerel, 67087 Strasbourg, France; j.schneider@inoviem.com (J.S.); christine.schaeffer@unistra.fr (C.S.-R.)

* Correspondence: mary-lorene.goddard@uha.fr (M.-L.G.); julie.chong@uha.fr (J.C.); Tel.: +33-3-89-33-67-69 (M.-L.G.)

† Present Address: Department of Plant Pathology, University of Stellenbosch, Private Bag X1, Matieland, 7602, South Africa.

‡ Present Address: Inoviem Scientific, 850 Boulevard Sébastien Brand, 67400 Illkrich-Graffenstaden, France.

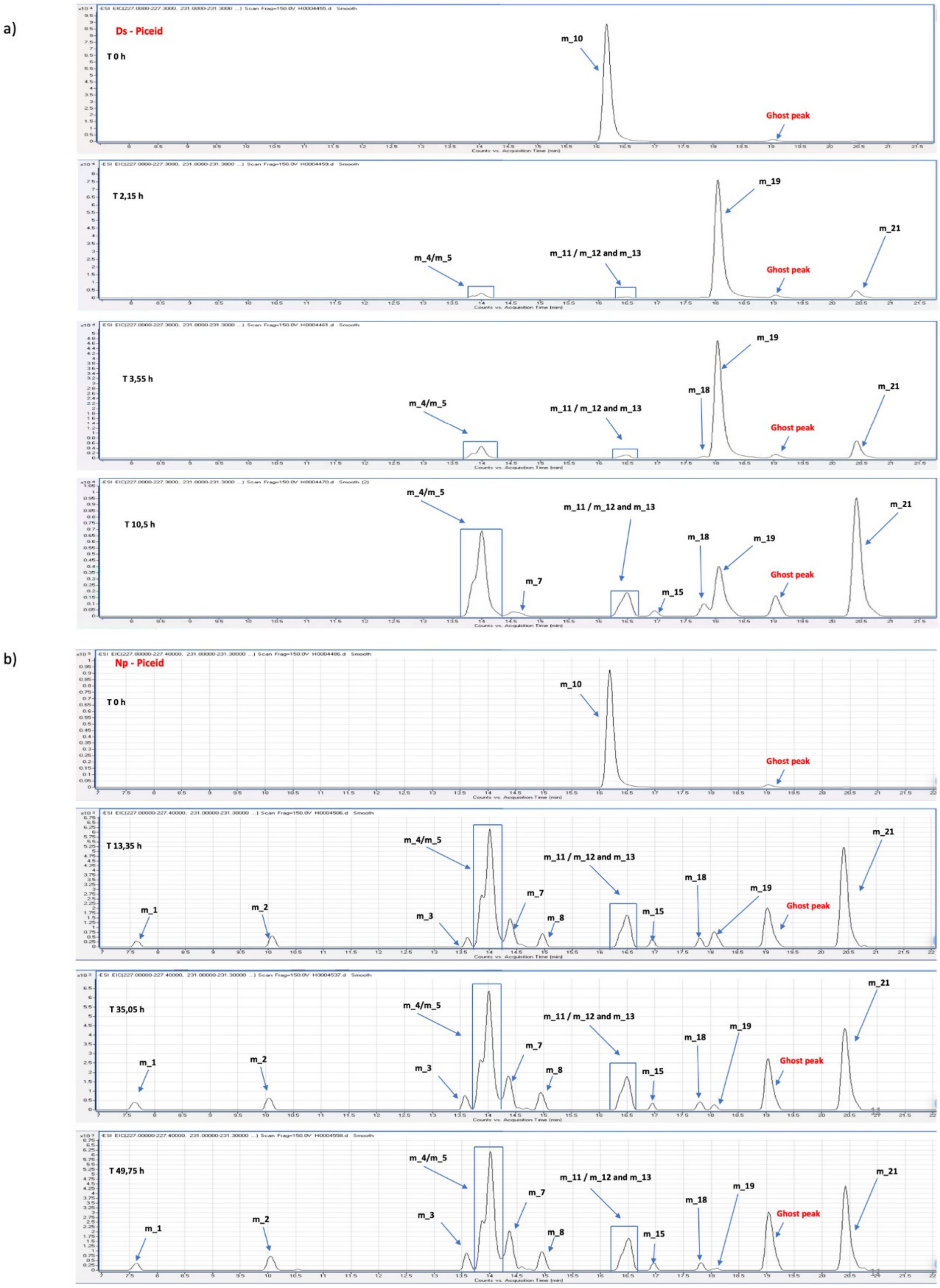

Figure S1. Extract Ion Chromatograms (EIC) obtained from piceid metabolization by (**a**) *D. seriata* and (**b**) *N. parvum* extracellular proteins at different time points.

a)

b)

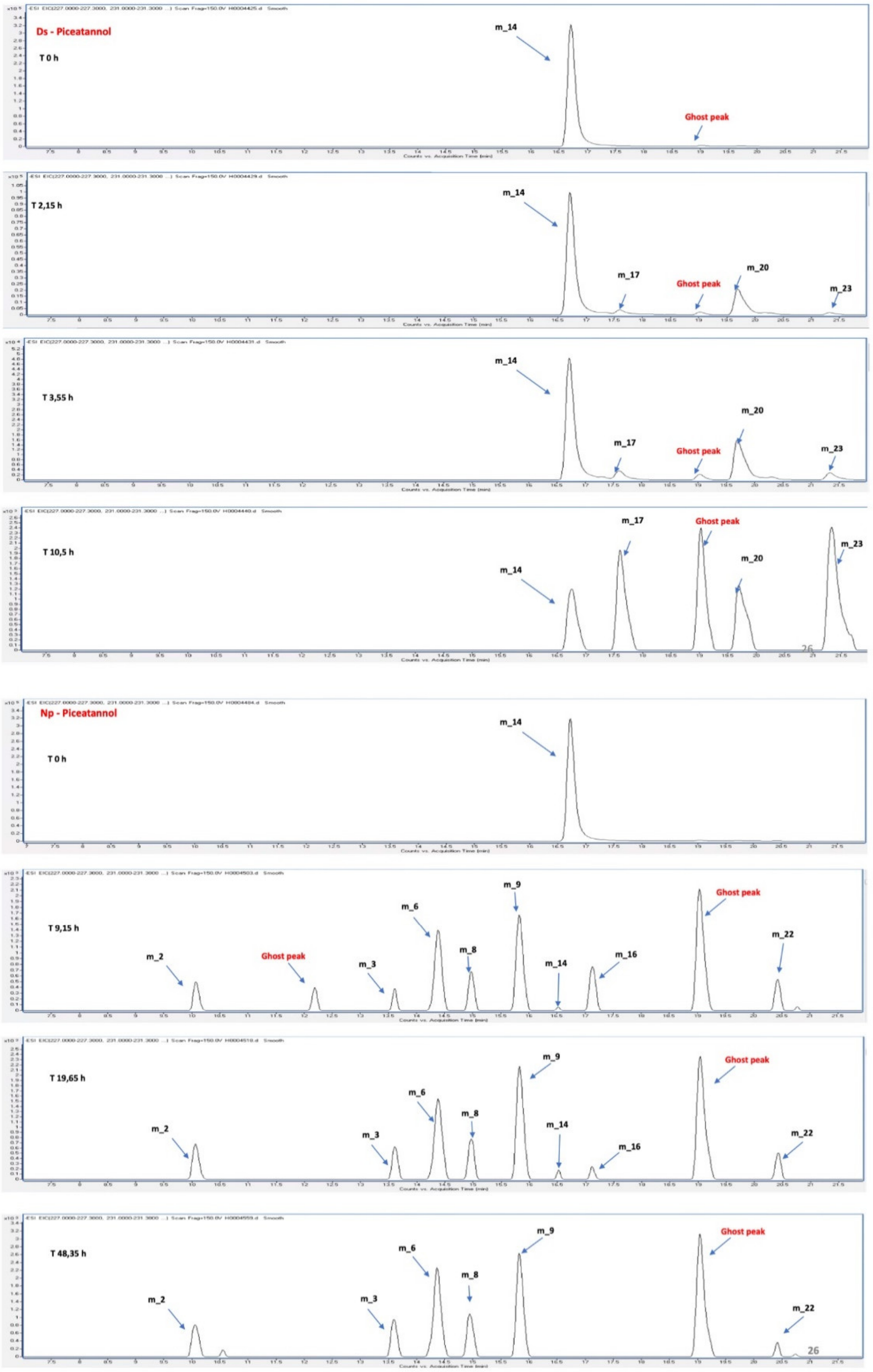

Figure S2. Extract Ion Chromatograms (EIC) obtained from piceatannol metabolization by (**a**) *D. seriata* and (**b**) *N. parvum* extracellular proteins at different time points.

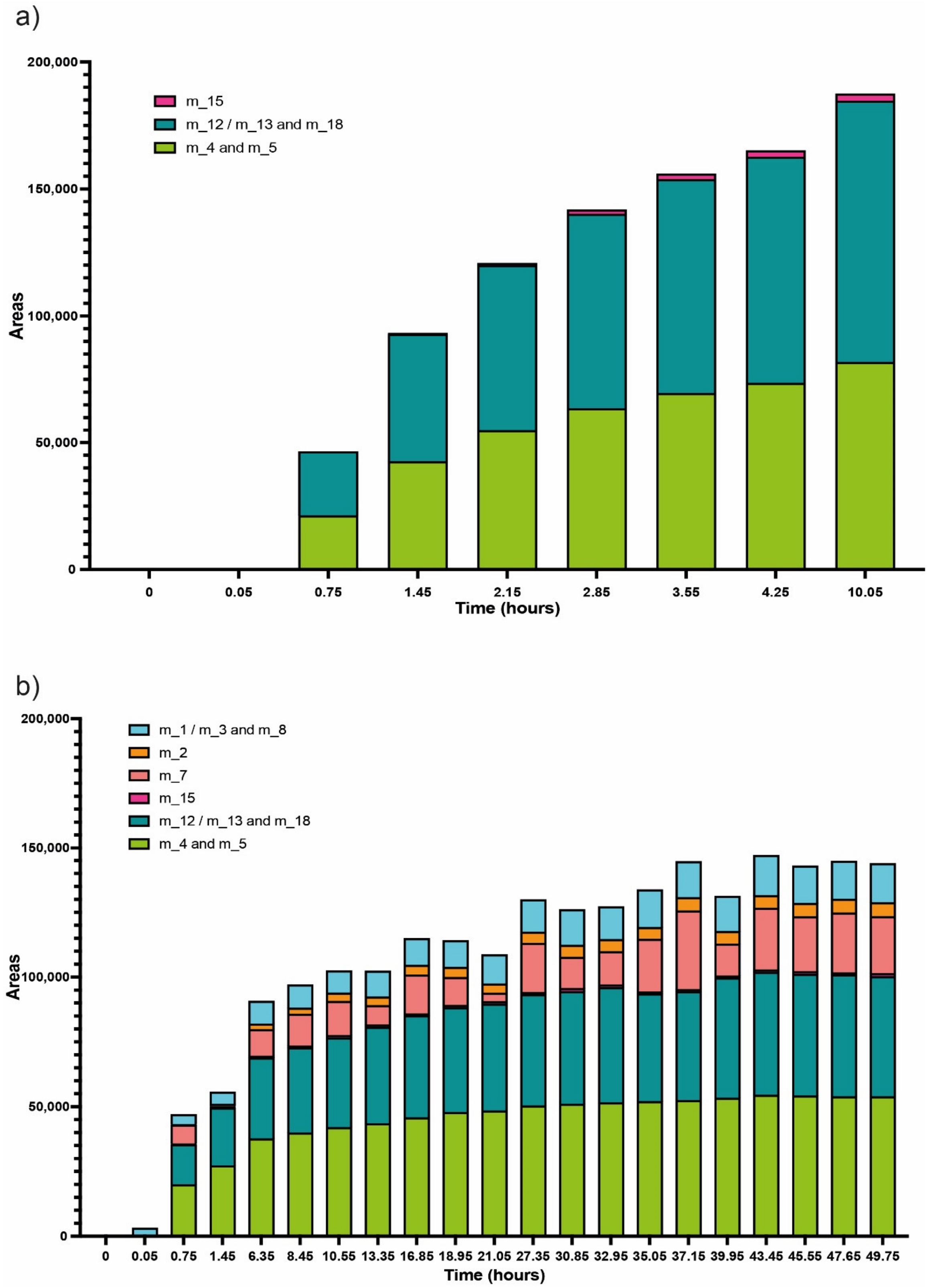

Figure S3. Putative metabolization products obtained during kinetic monitoring of trans-piceid (5 µg/mL) metabolization in sodium acetate buffer by total extracellular protein extract from (**a**) *D. seriata* (200 µg/mL) and (**b**) *N. parvum* (1 mg/mL). Mean was calculated over three biological replicates each comprising two technical replicates (n = 6).

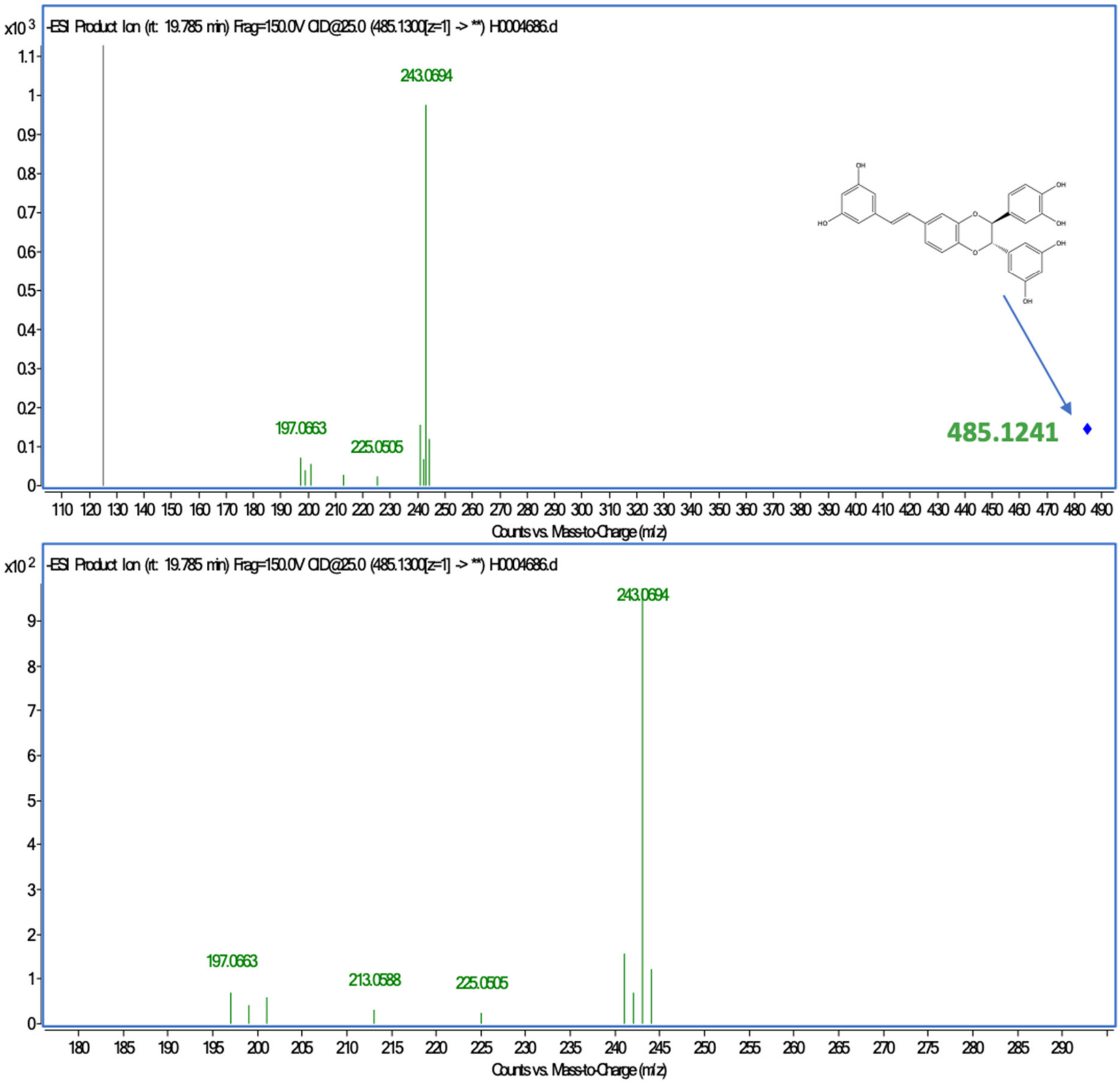

Figure S4. MS-MS spectrum for putative identification of Cassigarol E with Mass Hunter (Agilent Technologies, Santa Clara, CA, USA).

Table S1. List of N. parvum secreted proteins according to the culture medium.

Entry Name	Gene Name	Identified in *N. parvum* Malt	Identified in *N. parvum* Malt+Wood	Molecular Weight	Enzyme Class
R1H3P0_BOTPV	UCRNP2_156	Yes	Yes	36908	Hydrolase
R1H3B2_BOTPV	UCRNP2_282	Yes	Yes	100513	Hydrolase
R1H2Y1_BOTPV	UCRNP2_405	Yes	Yes	49829	Hydrolase
R1H1X1_BOTPV	UCRNP2_795	Yes	Yes	110578	Hydrolase
R1H0S9_BOTPV	UCRNP2_1147	Yes	Yes	74639	Hydrolase
R1H0M7_BOTPV	UCRNP2_1242	Yes	Yes	42532	Hydrolase
R1GZU2_BOTPV	UCRNP2_1593	Yes	Yes	33329	Hydrolase
R1GZQ9_BOTPV	UCRNP2_1648	Yes	Yes	39323	Hydrolase
R1GZN3_BOTPV	UCRNP2_1674	Yes	Yes	48044	Hydrolase
R1GXI5_BOTPV	UCRNP2_2427	Yes	Yes	37825	Hydrolase
R1GWK1_BOTPV	UCRNP2_2762	Yes	Yes	79702	Hydrolase

R1GVI3_BOTPV	UCRNP2_3149	Yes	Yes	57551	Hydrolase
R1GUX3_BOTPV	UCRNP2_1073	Yes	Yes	61964	Hydrolase
R1GUW7_BOTPV	UCRNP2_3286	Yes	Yes	43262	Hydrolase
R1GTC8_BOTPV	UCRNP2_3892	Yes	Yes	64829	Hydrolase
R1GT23_BOTPV	UCRNP2_4011	Yes	Yes	57761	Hydrolase
R1GSR4_BOTPV	UCRNP2_1891	Yes	Yes	36394	Hydrolase
R1GSK1_BOTPV	UCRNP2_1980	Yes	Yes	61094	Hydrolase
R1GS00_BOTPV	UCRNP2_2220	Yes	Yes	75439	Hydrolase
R1GRI8_BOTPV	UCRNP2_2356	Yes	Yes	36453	Hydrolase
R1GRI6_BOTPV	UCRNP2_4663	Yes	Yes	61499	Hydrolase
R1GQ85_BOTPV	UCRNP2_2804	No	Yes	51011	Hydrolase
R1GPX5_BOTPV	UCRNP2_2898	Yes	Yes	27087	Hydrolase
R1GPT3_BOTPV	UCRNP2_2931	Yes	Yes	91543	Hydrolase
R1GPA2_BOTPV	UCRNP2_5425	Yes	Yes	56053	Hydrolase
R1GP89_BOTPV	UCRNP2_5424	Yes	Yes	47449	Hydrolase
R1GNJ6_BOTPV	UCRNP2_3307	Yes	Yes	60383	Hydrolase
R1GMR9_BOTPV	UCRNP2_5948	Yes	Yes	46108	Hydrolase
R1GMR8_BOTPV	UCRNP2_3675	Yes	Yes	86835	Hydrolase
R1GM42_BOTPV	UCRNP2_6229	Yes	Yes	41788	Hydrolase
R1GLG1_BOTPV	UCRNP2_691	Yes	Yes	68532	Hydrolase
R1GK17_BOTPV	UCRNP2_1096	Yes	Yes	42117	Hydrolase
R1GJH9_BOTPV	UCRNP2_7128	Yes	Yes	25771	Hydrolase
R1GIR6_BOTPV	UCRNP2_7424	Yes	Yes	55869	Hydrolase
R1GIC9_BOTPV	UCRNP2_5210	Yes	Yes	46266	Hydrolase
R1GHS1_BOTPV	UCRNP2_1987	Yes	Yes	44649	Hydrolase
R1GHM7_BOTPV	UCRNP2_2062	Yes	Yes	41869	Hydrolase
R1GHF4_BOTPV	UCRNP2_2168	Yes	Yes	56748	Hydrolase
R1GGZ6_BOTPV	UCRNP2_8019	Yes	Yes	40494	Hydrolase
R1GGQ9_BOTPV	UCRNP2_8156	Yes	Yes	38051	Hydrolase
R1GGK2_BOTPV	UCRNP2_5863	Yes	Yes	83544	Hydrolase
R1GFF8_BOTPV	UCRNP2_6288	Yes	Yes	65834	Hydrolase
R1GF72_BOTPV	UCRNP2_8712	Yes	Yes	75040	Hydrolase
R1GF60_BOTPV	UCRNP2_8732	Yes	Yes	52147	Hydrolase
R1GEY0_BOTPV	UCRNP2_3078	Yes	Yes	77417	Hydrolase
R1GET5_BOTPV	UCRNP2_6495	Yes	Yes	47228	Hydrolase
R1GEP3_BOTPV	UCRNP2_6533	Yes	Yes	51789	Hydrolase
R1GE12_BOTPV	UCRNP2_3376	Yes	Yes	31910	Hydrolase
R1GDJ0_BOTPV	UCRNP2_6952	Yes	Yes	37646	Hydrolase
R1GDD4_BOTPV	UCRNP2_3654	No	Yes	60065	Hydrolase
R1GD62_BOTPV	UCRNP2_3810	No	Yes	32071	Hydrolase
R1GD52_BOTPV	UCRNP2_9440	Yes	Yes	43378	Hydrolase
R1GCR8_BOTPV	UCRNP2_7276	Yes	Yes	98101	Hydrolase
R1GCQ7_BOTPV	UCRNP2_3893	Yes	Yes	61244	Hydrolase
R1GBW7_BOTPV	UCRNP2_4217	Yes	Yes	41020	Hydrolase
R1GAY7_BOTPV	UCRNP2_10259	Yes	Yes	60835	Hydrolase
R1GAI1_BOTPV	UCRNP2_4714	Yes	Yes	34758	Hydrolase
R1GAE5_BOTPV	UCRNP2_4781	Yes	Yes	61141	Hydrolase
R1G9V6_BOTPV	UCRNP2_4913	Yes	Yes	38806	Hydrolase

R1G9E1_BOTPV	UCRNP2_5072	Yes	Yes	47146	Hydrolase
R1G8D0_BOTPV	UCRNP2_8897	Yes	Yes	25616	Hydrolase
R1G8C1_BOTPV	UCRNP2_5439	Yes	Yes	39212	Hydrolase
R1G7G3_BOTPV	UCRNP2_5817	Yes	Yes	44118	Hydrolase
R1G776_BOTPV	UCRNP2_9363	Yes	Yes	60904	Hydrolase
R1G6Y8_BOTPV	UCRNP2_9407	Yes	Yes	34374	Hydrolase
R1G6D0_BOTPV	UCRNP2_9564	Yes	Yes	43070	Hydrolase
R1G5Y4_BOTPV	UCRNP2_9853	Yes	Yes	56778	Hydrolase
R1G5R2_BOTPV	UCRNP2_9781	Yes	Yes	31462	Hydrolase
R1G0U7_BOTPV	UCRNP2_8284	No	Yes	76268	Hydrolase
R1FVA2_BOTPV	UCRNP2_10321	Yes	Yes	70539	Hydrolase
R1FV38_BOTPV	UCRNP2_10352	Yes	Yes	62478	Hydrolase
R1EYI5_BOTPV	UCRNP2_364	Yes	Yes	20173	Hydrolase
R1EXV1_BOTPV	UCRNP2_612	Yes	Yes	88843	Hydrolase
R1EXU8_BOTPV	UCRNP2_626	Yes	Yes	60373	Hydrolase
R1EVA1_BOTPV	UCRNP2_1506	Yes	Yes	51365	Hydrolase
R1EUP8_BOTPV	UCRNP2_1879	Yes	Yes	48877	Hydrolase
R1EUG4_BOTPV	UCRNP2_1970	Yes	Yes	74938	Hydrolase
R1EUD5_BOTPV	UCRNP2_1812	Yes	Yes	107169	Hydrolase
R1EU52_BOTPV	UCRNP2_2106	Yes	Yes	51345	Hydrolase
R1EQB5_BOTPV	UCRNP2_3458	No	Yes	51200	Hydrolase
R1EQ49_BOTPV	UCRNP2_3524	Yes	Yes	46705	Hydrolase
R1EP88_BOTPV	UCRNP2_3629	Yes	Yes	84093	Hydrolase
R1EKL4_BOTPV	UCRNP2_5185	Yes	Yes	57577	Hydrolase
R1EJQ6_BOTPV	UCRNP2_5270	Yes	Yes	66967	Hydrolase
R1EJE5_BOTPV	UCRNP2_5592	Yes	Yes	33879	Hydrolase
R1EJ08_BOTPV	UCRNP2_5476	Yes	Yes	35296	Hydrolase
R1EIF4_BOTPV	UCRNP2_5965	Yes	Yes	31915	Hydrolase
R1EHV9_BOTPV	UCRNP2_5888	Yes	Yes	56459	Hydrolase
R1EGV3_BOTPV	UCRNP2_6272	Yes	Yes	49745	Hydrolase
R1EGF8_BOTPV	UCRNP2_6392	No	Yes	67626	Hydrolase
R1EFS2_BOTPV	UCRNP2_6649	Yes	Yes	57590	Hydrolase
R1EFN6_BOTPV	UCRNP2_6948	Yes	Yes	85359	Hydrolase
R1EFK4_BOTPV	UCRNP2_6745	Yes	Yes	32419	Hydrolase
R1EF79_BOTPV	UCRNP2_7122	Yes	Yes	62810	Hydrolase
R1EEC1_BOTPV	UCRNP2_7446	Yes	Yes	108544	Hydrolase
R1EDI8_BOTPV	UCRNP2_7779	Yes	Yes	37269	Hydrolase
R1EDH3_BOTPV	UCRNP2_7797	Yes	Yes	34892	Hydrolase
R1EBK2_BOTPV	UCRNP2_8434	Yes	Yes	43902	Hydrolase
R1EAR3_BOTPV	UCRNP2_8796	Yes	Yes	82417	Hydrolase
R1EAH5_BOTPV	UCRNP2_8476	No	Yes	44478	Hydrolase
R1E6Y2_BOTPV	UCRNP2_10119	Yes	Yes	59873	Hydrolase
R1E681_BOTPV	UCRNP2_10067	Yes	Yes	41350	Hydrolase
R1E623_BOTPV	UCRNP2_10453	Yes	Yes	40346	Hydrolase

R1E5V7_BOTPV	UCRNP2_10238	Yes	Yes	47907	Hydrolase
R1H3Z2_BOTPV	UCRNP2_37	Yes	No	53261	Hydrolase
R1GVP5_BOTPV	UCRNP2_3045	Yes	No	42418	Hydrolase
R1GVK7_BOTPV	UCRNP2_3108	Yes	No	61218	Hydrolase
R1GU94_BOTPV	UCRNP2_3593	Yes	No	49345	Hydrolase
R1GT47_BOTPV	UCRNP2_3967	Yes	No	37679	Hydrolase
R1GP56_BOTPV	UCRNP2_3148	Yes	No	58683	Hydrolase
R1GMY2_BOTPV	UCRNP2_157	Yes	No	62020	Hydrolase
R1GMU2_BOTPV	UCRNP2_5929	Yes	No	84731	Hydrolase
R1GM11_BOTPV	UCRNP2_6259	Yes	No	39070	Hydrolase
R1GKX0_BOTPV	UCRNP2_862	Yes	No	66019	Hydrolase
R1GHJ3_BOTPV	UCRNP2_7843	Yes	No	58112	Hydrolase
R1GHG7_BOTPV	UCRNP2_7883	Yes	No	60096	Hydrolase
R1GHB2_BOTPV	UCRNP2_2217	Yes	No	65346	Hydrolase
R1GHA8_BOTPV	UCRNP2_5569	Yes	No	91841	Hydrolase
R1GG38_BOTPV	UCRNP2_2643	Yes	No	81042	Hydrolase
R1GEZ5_BOTPV	UCRNP2_3039	Yes	No	47901	Hydrolase
R1GD80_BOTPV	UCRNP2_3729	Yes	No	34812	Hydrolase
R1GCJ5_BOTPV	UCRNP2_7325	Yes	No	39463	Hydrolase
R1GC39_BOTPV	UCRNP2_4063	Yes	No	39581	Hydrolase
R1GBR8_BOTPV	UCRNP2_7651	Yes	No	40706	Hydrolase
R1GBC9_BOTPV	UCRNP2_4379	Yes	No	63192	Hydrolase
R1GB50_BOTPV	UCRNP2_10225	Yes	No	54235	Hydrolase
R1GAZ1_BOTPV	UCRNP2_10271	Yes	No	73679	Hydrolase
R1G8X2_BOTPV	UCRNP2_8676	Yes	No	25831	Hydrolase
R1G8H3_BOTPV	UCRNP2_5421	Yes	No	171488	Hydrolase
R1G7D0_BOTPV	UCRNP2_5851	Yes	No	105043	Hydrolase
R1G5D2_BOTPV	UCRNP2_6628	Yes	No	45626	Hydrolase
R1G324_BOTPV	UCRNP2_7463	Yes	No	84322	Hydrolase
R1G2T0_BOTPV	UCRNP2_7553	Yes	No	48647	Hydrolase
R1G0M1_BOTPV	UCRNP2_8337	Yes	No	60702	Hydrolase
R1FWJ1_BOTPV	UCRNP2_9839	Yes	No	51950	Hydrolase
R1EZX9_BOTPV	UCRNP2_13	Yes	No	46166	Hydrolase
R1EX46_BOTPV	UCRNP2_980	Yes	No	54903	Hydrolase
R1EWH5_BOTPV	UCRNP2_1261	Yes	No	62216	Hydrolase
R1EW80_BOTPV	UCRNP2_1160	Yes	No	44814	Hydrolase
R1EUA7_BOTPV	UCRNP2_1833	Yes	No	56830	Hydrolase
R1ET60_BOTPV	UCRNP2_2462	Yes	No	42724	Hydrolase
R1ERC6_BOTPV	UCRNP2_2876	Yes	No	42927	Hydrolase
R1EPE0_BOTPV	UCRNP2_3571	Yes	No	97038	Hydrolase
R1EP80_BOTPV	UCRNP2_3871	Yes	No	86510	Hydrolase
R1ENX6_BOTPV	UCRNP2_3973	Yes	No	59291	Hydrolase
R1EJ45_BOTPV	UCRNP2_5423	Yes	No	64761	Hydrolase
R1EGT1_BOTPV	UCRNP2_6531	Yes	No	31092	Hydrolase
R1ECV1_BOTPV	UCRNP2_7960	Yes	No	87056	Hydrolase
R1EBD7_BOTPV	UCRNP2_8492	Yes	No	38070	Hydrolase

R1E9R3_BOTPV	UCRNP2_8802	Yes	No	41490	Hydrolase
R1E9F8_BOTPV	UCRNP2_9287	Yes	No	69955	Hydrolase
R1E8Z9_BOTPV	UCRNP2_9401	Yes	No	123316	Hydrolase
R1E8S9_BOTPV	UCRNP2_9099	Yes	No	55477	Hydrolase
R1E8B6_BOTPV	UCRNP2_9610	Yes	No	45528	Hydrolase
R1E7W9_BOTPV	UCRNP2_9420	Yes	No	108086	Hydrolase
R1E7V5_BOTPV	UCRNP2_9444	Yes	No	61800	Hydrolase
R1E713_BOTPV	UCRNP2_10059	Yes	No	48432	Hydrolase
R1E6N5_BOTPV	UCRNP2_10275	Yes	No	46519	Hydrolase
R1GVC0_BOTPV	UCRNP2_3215	Yes	Yes	43748	Isomerase
R1GHN0_BOTPV	UCRNP2_5480	Yes	Yes	38083	Isomerase
R1GU70_BOTPV	UCRNP2_1378	Yes	Yes	42793	Lyase
R1GN84_BOTPV	UCRNP2_3453	No	Yes	25245	Lyase
R1E934_BOTPV	UCRNP2_9371	Yes	No	61924	Lyase
R1H382_BOTPV	UCRNP2_317	Yes	Yes	75606	Non enzymatic protein
R1GSH0_BOTPV	UCRNP2_4276	Yes	No	18262	Non enzymatic protein
R1GKT0_BOTPV	UCRNP2_4338	Yes	Yes	70839	Non enzymatic protein
R1GID5_BOTPV	UCRNP2_7520	Yes	Yes	57999	Non enzymatic protein
R1GIC8_BOTPV	UCRNP2_1786	Yes	Yes	16039	Non enzymatic protein
R1GHS9_BOTPV	UCRNP2_1979	Yes	Yes	35951	Non enzymatic protein
R1GF67_BOTPV	UCRNP2_8720	Yes	No	43044	Non enzymatic protein
R1G8H7_BOTPV	UCRNP2_8856	Yes	Yes	27337	Non enzymatic protein
R1G7H5_BOTPV	UCRNP2_9267	Yes	Yes	38776	Non enzymatic protein
R1G676_BOTPV	UCRNP2_9627	Yes	No	25673	Non enzymatic protein
R1G352_BOTPV	UCRNP2_7406	Yes	Yes	21962	Non enzymatic protein
R1G1Q3_BOTPV	UCRNP2_7919	Yes	No	14190	Non enzymatic protein
R1FVG4_BOTPV	UCRNP2_10235	Yes	Yes	45325	Non enzymatic protein
R1EWZ5_BOTPV	UCRNP2_892	Yes	Yes	49696	Non enzymatic protein
R1ETS0_BOTPV	UCRNP2_2255	Yes	Yes	46920	Non enzymatic protein
R1EPB0_BOTPV	UCRNP2_3869	Yes	No	18527	Non enzymatic protein
R1EIN7_BOTPV	UCRNP2_5605	Yes	No	32793	Non enzymatic protein

R1EGH3_BOTPV	UCRNP2_6648	Yes	Yes	76953	Non enzymatic protein
R1EE60_BOTPV	UCRNP2_7269	Yes	No	99248	Non enzymatic protein
R1E9V1_BOTPV	UCRNP2_8743	Yes	No	48901	Non enzymatic protein
R1E8I6_BOTPV	UCRNP2_9221	Yes	No	15362	Non enzymatic protein
R1E6W1_BOTPV	UCRNP2_10157	Yes	Yes	15359	Non enzymatic protein
R1E6C5_BOTPV	UCRNP2_10044	Yes	No	36560	Non enzymatic protein
R1G8U3_BOTPV	UCRNP2_8718	No	Yes	106910	Non enzymatic protein
R1EG56_BOTPV	UCRNP2_6496	No	Yes	28362	Non enzymatic protein
R1FV21_BOTPV	UCRNP2_10356	Yes	Yes	40357	Non enzymatic protein
R1EZ07_BOTPV	UCRNP2_343	Yes	Yes	41037	Non enzymatic protein
R1H3D6_BOTPV	UCRNP2_257	Yes	No	62395	Non enzymatic protein
R1GXL3_BOTPV	UCRNP2_2387	Yes	No	58449	Non enzymatic protein
R1GJC2_BOTPV	UCRNP2_7232	Yes	No	81077	Non enzymatic protein
R1GGV5_BOTPV	UCRNP2_8060	Yes	No	63091	Non enzymatic protein
R1G4B9_BOTPV	UCRNP2_10358	Yes	No	63193	Non enzymatic protein
R1G3P9_BOTPV	UCRNP2_7270	Yes	No	60777	Non enzymatic protein
R1FX65_BOTPV	UCRNP2_9580	Yes	No	51931	Non enzymatic protein
R1H3M7_BOTPV	UCRNP2_206	Yes	Yes	89115	Oxidoreductase
R1H231_BOTPV	UCRNP2_731	Yes	Yes	41115	Oxidoreductase
R1GQT1_BOTPV	UCRNP2_4898	Yes	Yes	38470	Oxidoreductase
R1GJT0_BOTPV	UCRNP2_7003	Yes	Yes	32232	Oxidoreductase
R1GB74_BOTPV	UCRNP2_10186	Yes	Yes	97034	Oxidoreductase
R1FVG2_BOTPV	UCRNP2_10242	Yes	Yes	63969	Oxidoreductase
R1ESA8_BOTPV	UCRNP2_2536	Yes	Yes	51681	Oxidoreductase
R1EKA6_BOTPV	UCRNP2_4996	Yes	Yes	64668	Oxidoreductase
R1EIT8_BOTPV	UCRNP2_5540	Yes	Yes	54791	Oxidoreductase
R1EGN1_BOTPV	UCRNP2_6594	Yes	Yes	60106	Oxidoreductase
R1EFL7_BOTPV	UCRNP2_6949	Yes	Yes	40563	Oxidoreductase
R1E7Q5_BOTPV	UCRNP2_9481	Yes	Yes	71393	Oxidoreductase
R1H000_BOTPV	UCRNP2_1480	Yes	No	67920	Oxidoreductase
R1GWD3_BOTPV	UCRNP2_490	Yes	No	78587	Oxidoreductase

R1GT20_BOTPV	UCRNP2_1824	Yes	No	58241	Oxidoreductase
R1GHB3_BOTPV	UCRNP2_5587	Yes	No	87357	Oxidoreductase
R1GB06_BOTPV	UCRNP2_4485	Yes	No	56308	Oxidoreductase
R1GAS7_BOTPV	UCRNP2_10354	Yes	No	64354	Oxidoreductase
R1GAE2_BOTPV	UCRNP2_8121	Yes	No	61247	Oxidoreductase
R1EF46_BOTPV	UCRNP2_6896	Yes	No	46234	Oxidoreductase
R1EF40_BOTPV	UCRNP2_6897	Yes	No	70583	Oxidoreductase
R1ED71_BOTPV	UCRNP2_7554	Yes	No	70157	Oxidoreductase
R1EAK6_BOTPV	UCRNP2_8837	Yes	No	30757	Oxidoreductase
R1GMD5_BOTPV	UCRNP2_3761	Yes	No	35620	Transferase
R1GGJ5_BOTPV	UCRNP2_8221	Yes	No	57479	Transferase
R1GAU0_BOTPV	UCRNP2_4537	Yes	No	48367	Transferase
R1GRM4_BOTPV	UCRNP2_2362	Yes	Yes	22861	Uncharacterized
R1GMX5_BOTPV	UCRNP2_3570	Yes	Yes	20526	Uncharacterized
R1GLJ5_BOTPV	UCRNP2_6391	Yes	Yes	44361	Uncharacterized
R1FZJ6_BOTPV	UCRNP2_8740	Yes	Yes	19798	Uncharacterized
R1H157_BOTPV	UCRNP2_994	Yes	No	22009	Uncharacterized
R1GYB0_BOTPV	UCRNP2_2169	Yes	No	22365	Uncharacterized
R1GU06_BOTPV	UCRNP2_3690	Yes	No	38129	Uncharacterized
R1GL84_BOTPV	UCRNP2_761	Yes	No	54276	Uncharacterized
R1GK87_BOTPV	UCRNP2_6872	Yes	No	58715	Uncharacterized
R1GIN0_BOTPV	UCRNP2_7431	Yes	No	29136	Uncharacterized
R1G7F7_BOTPV	UCRNP2_9259	Yes	No	26935	Uncharacterized
R1G0W5_BOTPV	UCRNP2_8261	Yes	No	29883	Uncharacterized
R1G013_BOTPV	UCRNP2_8533	Yes	No	39667	Uncharacterized
R1FV17_BOTPV	UCRNP2_10381	Yes	No	19147	Uncharacterized
R1EXH8_BOTPV	UCRNP2_876	Yes	No	32463	Uncharacterized
R1EUX4_BOTPV	UCRNP2_1811	Yes	No	13073	Uncharacterized
R1EFP1_BOTPV	UCRNP2_6940	Yes	No	13297	Uncharacterized
R1EBX7_BOTPV	UCRNP2_7984	Yes	No	42669	Uncharacterized

Table S2. *N. parvum* secreted proteins identified according to the culture medium (malt, malt with wood and both).

	**Malt**	**Malt + Wood**	**Both**
Hydrolases	54	7	97
Isomerases	0	0	2
Lyases	1	1	1
Oxidoreductases	11	0	12
Transferases	3	0	0
Ligases	0	0	0
Uncharacterized	14	0	4
Non-enzymatic proteins	17	2	15

Table S3. List of *D. seriata* secreted proteins according to the culture medium.

**Entry Name**	**Gene Name**	**Found in *D. seriata* Malt**	**Found in *D. seriata* Malt+Wood**	**Molecular Weigth**	**Enzyme Class**

A0A0G2EU45_9PEZI	UCDDS831_g01842	No	Yes	23197	Hydrolase
A0A0G2G871_9PEZI	UCDDS831_g05153	No	Yes	81651	Hydrolase
A0A0G2DZL6_9PEZI	UCDDS831_g07378	No	Yes	90322	Hydrolase
A0A0G2EFN8_9PEZI	UCDDS831_g04332	No	Yes	22835	Hydrolase
A0A0G2GWI0_9PEZI	UCDDS831_g04419	Yes	No	62255	Hydrolase
A0A0G2G1W8_9PEZI	UCDDS831_g06213	No	Yes	131493	Ligase
A0A0G2E8U8_9PEZI	UCDDS831_g05366	Yes	Yes	34301	Non enzymatic protein
A0A0G2DVF8_9PEZI	BK809_0002194<br>UCDDS831_g08038	No	Yes	14194	Non enzymatic protein
A0A0G2GBY8_9PEZI	BK809_0002193<br>UCDDS831_g08039	No	Yes	14884	Non enzymatic protein
A0A0G2GFP8_9PEZI	UCDDS831_g03603	No	Yes	16968	Non enzymatic protein
A0A0G2G3T6_9PEZI	BK809_0003196<br>UCDDS831_g05962	No	Yes	20951	Non enzymatic protein
A0A0G2GS40_9PEZI	UCDDS831_g05274	No	Yes	43206	Non enzymatic protein
A0A0G2EVA1_9PEZI	BK809_0007516<br>UCDDS831_g01541	No	Yes	36669	Non enzymatic protein
A0A0G2ELD8_9PEZI	UCDDS831_g03391	No	Yes	38772	Non enzymatic protein
A0A0G2GPT0_9PEZI	BK809_0000942<br>UCDDS831_g05765	No	Yes	23769	Non enzymatic protein
A0A0G2EVK6_9PEZI	BK809_0007423<br>UCDDS831_g01651	No	Yes	59802	Non enzymatic protein
A0A0G2F1Q4_9PEZI	UCDDS831_g00403	No	Yes	34541	Non enzymatic protein
A0A0G2HK23_9PEZI	UCDDS831_g00085	No	Yes	55359	Non enzymatic protein
A0A0G2G5D2_9PEZI	BK809_0003014<br>UCDDS831_g05610	No	Yes	53928	Oxidoreductase
A0A0G2G355_9PEZI	UCDDS831_g06080	No	Yes	72503	Oxidoreductase
A0A0G2H0Y5_9PEZI	UCDDS831_g03635	No	Yes	48577	Oxidoreductase
A0A0G2EQU2_9PEZI	UCDDS831_g02257	No	Yes	36014	Oxidoreductase
A0A0G2GV48_9PEZI	UCDDS831_g04662	No	Yes	34432	Oxidoreductase
A0A0G2EAQ5_9PEZI	UCDDS831_g05258	No	Yes	65143	Oxidoreductase
A0A0G2DYL4_9PEZI	UCDDS831_g07336	Yes	Yes	41616	Transferase
A0A0G2F325_9PEZI	UCDDS831_g00084	No	Yes	79876	Transferase
A0A0G2GYA2_9PEZI	BK809_0004243<br>UCDDS831_g00503	No	Yes	16702	Transferase

Table S4. *D. seriata* secreted proteins identified according to the culture medium (malt, malt with wood and both).

	**Malt**	**Malt + Wood**	**Both**
Hydrolases	1	4	0
Isomerases	0	0	0
Lyases	0	0	0
Oxidoreductases	0	6	0
Transferases	0	2	1
Ligases	0	1	0
Uncharacterized	0	0	0
Non-enzymatic proteins	0	11	1

Table S5. List of compounds detected during trans-piceid, trans-resveratrol and trans-piceatannol metabolization.

Feature Name	RT (min)	Molecular Formula	Monoisotopic Mass	Calculated [M - H]- m/z	Measured [M - H]- m/z	│Δppm│	Base Peak	Assigned Compound	λ max (nm)	MS/MS Fragments [a]
m_1	7.63	$C_{14}H_{12}O_4$	244.07356	243.06628	243.06346	9.97	[M - H]-	[c]piceatannol isomer		
m_2	10.06				231.13226		[M - H]-			
m_3	13.60				243.16786		[M - H]-			
m_4	13.99	$C_{28}H_{24}O_7$	472.15220	471.14493	471.14371	2.59	[M - H]-	[a]Leachianol G	198, 232, 280	377.1014; 349.1410; 255.0857; 121.0374
m_5	14.15	$C_{28}H_{24}O_7$	472.15220	471.14493	471.14418	1.69	[M - H]-	[a]Leachianol F	196, 218	377.1043; 349.0979; 255.0857; 121.0386
m_6	14.43				243.16826		[M - H]-			
m_7	14.54	$C_{28}H_{24}O_7$	472.15220	471.14493	471.14206	5.87	[M - H]-	[b]restrytisol A	196, 217, 276	377.1047; 349.0982; 255.0857; 121.0384 @ 10 eV
m_8	14.97				243.16776		[M - H]-		192, 208	
m_9	15.82				231.06266	15,68	[M - H]-			
m_10	16.19	$C_{20}H_{22}O_8$	390.13147	389.2419	389.12196	5.74	[M - H]-	[a]*trans*-piceid	198, 218, 312	
m_11	16.47	$C_{28}H_{22}O_6$	454.14164	453.13436	453.13436	4.47	[M - H]-	[a]pallidol	202, 286	359.0923; 265.0582; 264.4424; 93.0423
m_12	16.54	$C_{28}H_{24}O_7$	472.15220	471.14493	471,14727	4.96	[M - H]-	[c]oxidized resveratrol dimer		
m_13	16.69	$C_{28}H_{24}O_7$	472.15220	471.14493	471,14697	4.32	[M - H]-	[c]oxidized resveratrol dimer		
m_14	16.73	$C_{14}H_{12}O_4$	244.07356	243.06628	243.06656	1.14	[M - H]-	[a]*trans*-piceatannol	222, 322	201.0527; 199.0723; 175.0734; 173.0581; 172.0501; 159.0426; 135.0440; 41.0041
m_15	16.96	$C_{29}H_{26}O_7$	486.16785	485.16058	485.15906	3.13	[M - H]-	[C]methoxylated resveratrol dimer		
m_16	17.12				231.06373	11,05	[M - H]-			
m_17	17.60	$C_{28}H_{22}O_8$	486.13147	485.12419	485.12086	6.87	[M - H]-	[c]piceatannol dimer		

m_18	17.80	$C_{28}H_{24}O_7$	472.15220	471.14493	471.14286	4.17	$[M - H]^-$	[c]oxidized resveratrol dimer	198, 218	
m_19	18.05	$C_{14}H_{12}O_3$	228.07864	227.07137	227.07138	0.05	$[M - H]^-$	[a]*trans*-resveratrol	220, 306	164.9418; 119.0411; 109.4032
m_20	19.69	$C_{28}H_{22}O_8$	486.13147	485.12419	485.12126	6.05	$[M - H]^-$	[b]Cassigarol E	198, 218	148.9555 ; 197.0615 ; 201.0560 ; 213.0555 ; 223.0441 ; 241.0534 ; 243.0696 ; 244.0733
m_21	20.42	$C_{28}H_{22}O_6$	454.14164	453.13436	453.13497	1.34	$[M - H]^-$	[a]*trans*-δ -viniferin	202, 222, 310	385.1347; 369.1079; 359.0865; 347.0872; 333.0713
m_22	20.43				231.11856		$[M - H]^-$			
m_23	21.33	$C_{28}H_{20}O_8$	484.11582	483.10854	483.10576	5.76	$[M - H]^-$	[c]piceatannol dimer		

All MS/MS spectra were recorded at 25 eV otherwise the collision energy is specified after the symbol @. [a] indicates metabolites with confirmed identification by standard, [b] indicates putative annotation by MS/MS and UV, [c] indicates non-characterized metabolities.
